# Prophages encode phage-defense systems with cognate self-immunity

**DOI:** 10.1016/j.chom.2021.09.002

**Published:** 2021-11-10

**Authors:** Siân V. Owen, Nicolas Wenner, Charles L. Dulberger, Ella V. Rodwell, Arthur Bowers-Barnard, Natalia Quinones-Olvera, Daniel J. Rigden, Eric J. Rubin, Ethan C. Garner, Michael Baym, Jay C.D. Hinton

**Affiliations:** 1 Department of Biomedical Informatics and Laboratory of Systems Pharmacology, Harvard Medical School, Boston, MA, USA; 2 Institute of Infection, Veterinary & Ecological Sciences, University of Liverpool, Liverpool, UK; 3 Biozentrum, University of Basel, Basel, Switzerland; 4 Department of Immunology and Infectious Diseases, Harvard T.H. Chan School of Public Health, Boston, MA, USA; 5 Department of Molecular and Cellular Biology, Harvard University, Boston, MA, USA; 6 Institute of Systems, Molecular and Integrative Biology, University of Liverpool, Liverpool, UK

**Keywords:** bacteria, prophage, phage defense, phage resistance, abortive infection, lysogeny, BstA, self-immunity, aba, superinfection

## Abstract

Temperate phages are pervasive in bacterial genomes, existing as vertically inherited islands termed prophages. Prophages are vulnerable to predation of their host bacterium by exogenous phages. Here, we identify BstA, a family of prophage-encoded phage-defense proteins in diverse Gram-negative bacteria. BstA localizes to sites of exogenous phage DNA replication and mediates abortive infection, suppressing the competing phage epidemic. During lytic replication, the BstA-encoding prophage is not itself inhibited by BstA due to self-immunity conferred by the anti-BstA (*aba*) element, a short stretch of DNA within the *bstA* locus. Inhibition of phage replication by distinct BstA proteins from *Salmonella*, *Klebsiella*, and *Escherichia* prophages is generally interchangeable, but each possesses a cognate *aba* element. The specificity of the *aba* element ensures that immunity is exclusive to the replicating prophage, preventing exploitation by variant BstA-encoding phages. The BstA protein allows prophages to defend host cells against exogenous phage attack without sacrificing the ability to replicate lytically.

## Highlights

• BstA is an abortive infection protein found in prophages of Gram-negative bacteria
• *aba*, a short DNA sequence within the *bstA* locus, acts as a self-immunity element
• *aba* gives BstA-encoding prophages immunity to BstA-driven abortive infection
• Variant BstA proteins have distinct and cognate *aba* elements

## Graphical abstract

**Figure:**
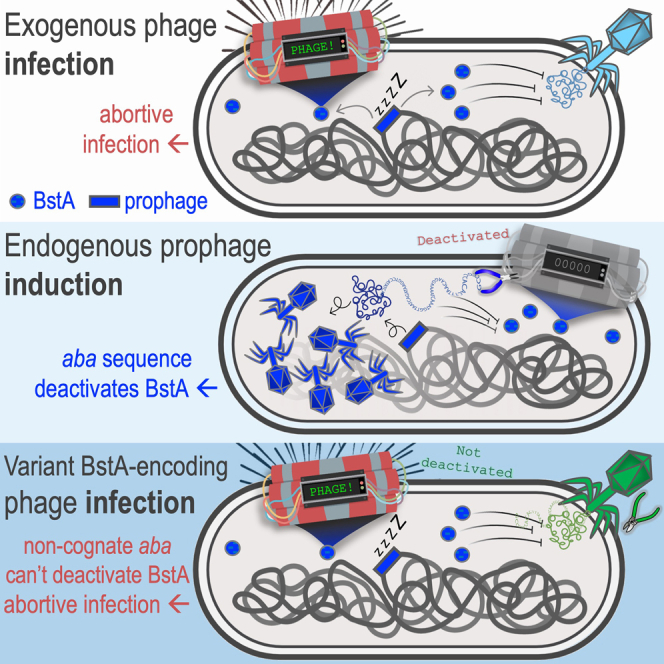

## Teaser

Prophages can encode abortive infection proteins that give their bacterial hosts population-level phage defense. Owen et al. show that some of these proteins contain internal self-immunity systems so that the prophage is not self-targeted by its own phage-defense protein.

## Introduction

The eternal battle between bacteria and their viruses (phages) has driven the evolution of a diverse array of phage-defense systems in bacteria (Bernheim and Sorek, 2020, Hampton et al., 2020, Rostøl and Marraffini, 2019, van Houte et al., 2016). Conversely, it is increasingly recognized that phages have evolved mechanisms to subvert these defense systems (Maxwell, 2017; Samson et al., 2013; Trasanidou et al., 2019).

Although the most intuitive form of phage defense involves the direct rescue of an infected cell, for example, by targeted degradation of phage nucleic acids by CRISPR-Cas or restriction modification systems, many phage-defense systems function solely at the population level. In a mechanism conceptually analogous to the pathogen-stimulated programmed cell death driven by the innate immune systems of higher organisms (Abedon, 2012), phage infection can be prevented from sweeping across populations, at the cost of the lives of infected cells. These population-level phage-defense systems are often grouped together under the umbrella term “abortive infection” (Abi) (Labrie et al., 2010; Lopatina et al., 2020) but actually represent diverse mechanisms to prevent phage replication and induce cell death (Bingham et al., 2000; Cohen et al., 2019; Fineran et al., 2009; Meeske et al., 2019; Pecota and Wood, 1996; Watson et al., 2019). Such mechanistic diversity and the high prevalence of abortive infection systems in nature emphasizes the selective advantage the Abi strategy imparts in the battle against phages (Benler and Koonin, 2020).

However, an important sub-plot in the bacteria-phage conflict is the widespread existence of so-called “temperate” or “lysogenic” phages within bacterial genomes. Temperate phages exist stably within the bacterial chromosome as latent, vertically inherited islands known as prophages. Crucially, to find new hosts, prophages must escape the bacterial genome and return to the lytic life cycle.

The prophage state imposes unique existential pressures because the fitness of the phage is indefinitely dependent on that of the host bacterium. To favor their own fitness, prophages frequently encode “moron” or “accessory” loci that modulate the biology of their host bacteria (Bondy-Denomy and Davidson, 2014; Cumby et al., 2012; Fortier and Sekulovic, 2013; Howard-Varona et al., 2017), a phenomenon likened to altruism (Shub, 1994).

An important trait conferred by prophages that can significantly increase bacterial fitness is resistance against bacteriophage attack. Indeed, recent work has suggested that prophage accessory genes are an underexplored reservoir of phage-defense systems (Bondy-Denomy and Davidson, 2014; Dedrick et al., 2017; Snyder, 1995).

Here, we report a phage-defense system driven by the BstA protein, which is encoded by prophages of diverse Gram-negative bacteria. When a bacterium harbors a BstA*-*encoding prophage, the BstA protein confers effective population-level defense against exogenous phage infection via abortive infection. The *bstA* locus includes an anti-BstA element, which suppresses the activity of the BstA protein to allow the native prophage to switch to a lytic lifestyle. We propose that this self-immunity mechanism has evolved to allow prophages to defend host cells from predatory phages without compromising their own lytic replication cycle.

## Results

### The BstA protein encoded by prophage BTP1 mediates phage defense

**Figure 1:**
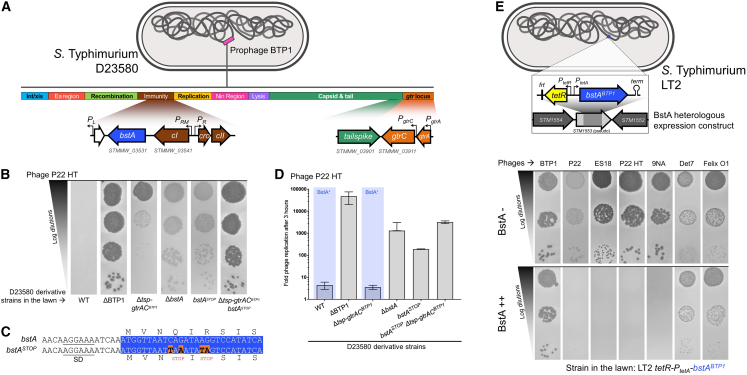
The *bstA* gene of prophage BTP1 confers phage defense (A) Genomic architecture of prophage BTP1 of *S.* Typhimurium D23580, according to Owen et al. (2020): the LPS modification genes *gtrAC*^*BTP1*^ (Kintz et al., 2015) and the immunity region carrying *bstA* (downstream of the *cI* repressor gene) are detailed. Bent arrows represent promoters. For reference purposes, the locus tags of important genes in this study in the D23580 reference genome (GenBank: FN424405.1) are shown. (B) Removal of prophage BTP1 from strain D23580 results in enhanced sensitivity to phage P22. Two BTP1 genes confer resistance to P22: *gtrAC* and *bstA*. Plaque assays were performed with phage P22 HT *105/1 int-201* (P22 HT) applied to lawns of *S.* Typhimurium D23580 WT or ΔBTP1, Δ*bstA*, *bstA*^*STOP*^, Δ*tsp-gtrAC*^*BTP1*^, and Δ*tsp-gtrAC*^*BTP1*^*bstA*^*STOP*^ mutants (strains JH3877, SSO-204, SSO-78, JH4287, and SNW431, respectively). The requirement for the inactivation of *tsp* is described in the STAR Methods. (C) The four nucleotide substitutions leading to two nonsense mutations in the *bstA*^*STOP*^ strain are indicated. SD indicates putative Shine-Dalgarno sequence of the *bstA* gene. The beginning of the *bstA* open reading frame is highlighted in blue. (D) Phage replication assays in liquid culture using P22 HT using the same D23580 derivative strains shown in the plaque assay in (B). Replication was measured 3 h post infection, and phages were enumerated on lawns of D23580 Δ*tsp-gtrAC bstA*^*STOP*^ (SNW431). Phage replication is presented as the mean of biological triplicates ± SD. (E) BstA protein confers phage defense in *S.* Typhimurium LT2. Phages P22, ES18, P22HT, and 9NA are inhibited by BstA. Phages Det7, Felix O1, and BTP1 are not affected by BstA expression. Plaque assays were carried out with the indicated *Salmonella* phages applied to lawns of LT2 *tetR-P*_*tetA*_*-bstA*^*BTP1*^ (JH4400) in the absence (BstA−) or the presence of the inducer anhydrotetracycline (AHT, BstA++). The *tetR*-*P*_*tetA*_-*bstA* insertion replacing a part of the *STM1553* pseudogene of strain JH4400 is schematized above: *tetR* encodes the tetracycline repressor that represses the *P*_*tetA*_ promoter in the absence of AHT induction, “*frt*” denotes the 84 nt scar sequence of pKD4, and the hairpin represents the native *bstA* Rho-independent terminator (*term*).

*Salmonella enterica* subsp. *enterica* serovar Typhimurium (hereafter *S.* Typhimurium) strain D23580 carries the ∼40 kb prophage BTP1 (Figure 1A) (Owen et al., 2017). An operon within BTP1, the *gtr* locus (*gtrAC*^*BTP1*^), confers resistance against phage P22 by chemically modifying the cellular lipopolysaccharide (LPS), the receptor for phage P22 (Kintz et al., 2015). Therefore, unsurprisingly, deleting the BTP1 prophage from strain D23580 (D23580 ΔBTP1) made the strain highly susceptible to infection by phage P22, confirming that resistance to phage P22 is conferred by BTP1 (Figure 1B). However, inactivation of the *gtr* locus of prophage BTP1 (D23580 Δ*tsp-gtrAC*^*BTP1*^) did not restore sensitivity to phage P22 to the level of D23580 ΔBTP1 (Figure 1B), suggesting the existence of a second BTP1-encoded phage-resistance system.

Previously, we used transcriptomics to discover that the *bstA* gene was highly expressed from prophage BTP1 during lysogeny, making it a candidate phage accessory gene (Owen et al., 2020). The *bstA* gene, encoded downstream of the prophage *cI* repressor locus, has been implicated phenotypically in both virulence and anti-virulence of *Salmonella* isolates, but no functional mechanism has been proposed (Herrero-Fresno et al., 2014, 2018; Spiegelhauer et al., 2020), and the BstA protein has not been characterized. We hypothesized that *bstA* was the second element in the BTP1 prophage that conferred defense against phage P22.

Consistent with this hypothesis, removal of the *bstA* gene from prophage BTP1 (D23580 Δ*bstA*) dramatically increased susceptibility to phage P22 (Figure 1B). To confirm that phage resistance was directly mediated by BstA protein, we introduced two stop codons into the beginning of the *bstA* coding sequence by exchanging 4 nucleotides (D23580 *bstA*^*STOP*^) (Figure 1C). D23580 *bstA*^*STOP*^ was highly susceptible to P22 phage, to the same level as D23580 Δ*bstA*, demonstrating that the BstA protein mediates defense against phage P22. Simultaneous deletion of the *gtr* locus and inactivation of the BstA protein (D23580 Δ*tsp-gtrAC*^*BTP1*^ *bstA*^*STOP*^) recapitulated the susceptibility to phage P22 achieved by deleting the entire BTP1 prophage (D23580 ΔBTP1), indicating that resistance to phage P22 was solely mediated by the *bstA* and *gtrAC* loci in prophage BTP1. These findings were reproduced by assaying the replication of phage P22 on the same strains in liquid culture, quantitatively demonstrating that reduction of plaque formation by BstA truly reflected suppression of phage replication (Figure 1D).

To investigate whether the defense function of the BstA protein depended on other elements from the BTP1 prophage, we constructed an inducible expression system in *S.* Typhimurium strain LT2, which does not contain the BTP1 prophage. LT2 is the type strain of *S.* Typhimurium and is natively susceptible to many phages, including P22 (McClelland et al., 2001). Expression of the BstA^BTP1^ protein in *S.* Typhimurium LT2 from within a neutral position on the chromosome (LT2 *tetR-P*_*tetA*_*-bstA*) conferred resistance to P22 and other phages, including ES18 and 9NA (Figures 1E and S1A).

While the induced expression of BstA^BTP1^ completely eliminated plaque formation of sensitive phages, at very high phage concentrations (10^9–10^ plaque forming units [PFU]/mL), these phages still produced clearing of the bacterial lawn (Figure S1B), which is consistent with an abortive infection mechanism of phage defense. The expression of the derivative containing two stop codons in the *bstA* coding sequence (*bstA*^*STOP*^) conferred no phage resistance, demonstrating again that defense is mediated by *bstA* at the protein level (Figure S1C). However, BstA did not mediate resistance against all of the tested phages. Det7, Felix O1, and notably, phage BTP1 (which encodes the *bstA* gene) were unaffected by the expression of BstA, both at the level of plaque assay and replication in liquid culture (Figures 1E and S1A). Induction of *bstA* or *bstA*^*STOP*^ expression in the absence of phage infection did not cause a detectable effect on cell growth rate, suggesting that overexpression of BstA^BTP1^ does not cause toxicity (Figure S1D). We were unable to detect any pattern in the characteristics or gene repertoire of phages that were sensitive or insensitive to BstA protein, which could relate to the mechanistic action of BstA protein.

### BstA represents a family of prophage-encoded phage-defense proteins in diverse Gram-negative bacteria

**Figure 2:**
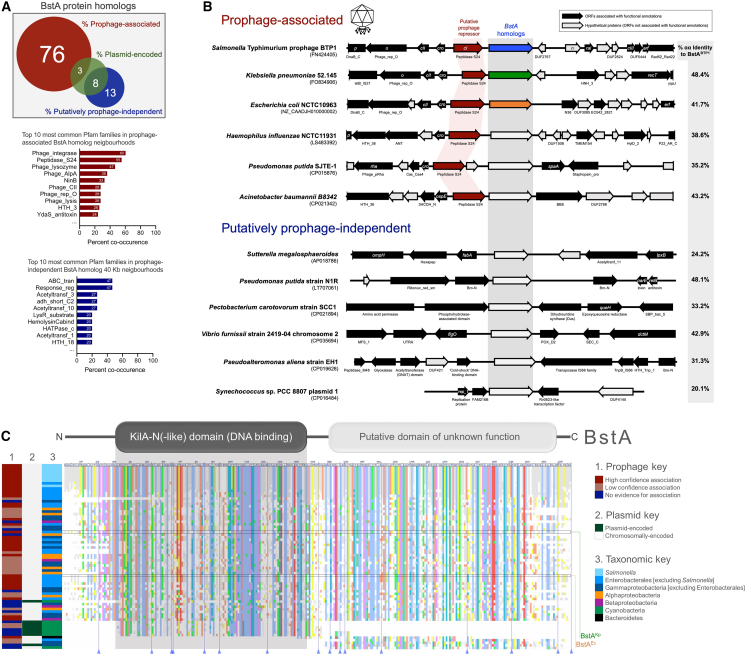
BstA homologs are found in diverse bacterial taxa and are frequently associated with prophages (A) A dataset of 72 BstA homologs representative of taxonomic diversity were manually curated and analyzed for prophage association based on the co-occurrence of phage-related Pfam domains in the 20 kb either side of each homolog (yielding a total 40 kb window) (Table S1). Homologs without co-occurring phage-related protein domains were assigned to be “putatively prophage independent.” A further subset of the BstA homologs were encoded on plasmids. The top ten most commonly co-occurring Pfam domains with prophage-associated and putatively prophage-independent BstA homologs are shown as bar graphs. (B) Gene maps showing the genetic context of a selection of 6 prophage-associated and 6 putatively prophage-independent BstA homologs (homologs indicated by the gray rectangle). Putative prophage repressor genes are highlighted in red. The top three BstA proteins from BTP1 (BstA^BTP1^, blue), *K. pneumoniae* 52.145 (BstA^Kp^, green), and *E. coli* NCTC10963 (BstA^Ec^, orange) are studied experimentally in later stages of this work and therefore are highlighted. Open reading frames associated with functional annotations are shown as solid black arrows, and functional gene names or Pfam domains are annotated. (C) An alignment of the 72 BstA protein homologs to BstA^BTP1^, with colors indicating amino acid conservation (Clustal color scheme). Alignment columns containing gaps relative to the reference sequence (BstA^BTP1^) have been collapsed and are indicated with blue lines and triangles at the base of the alignment (an expanded alignment can be found in Figure S2A). The position of BstA^Kp^ and BstA^Ec^ within the alignment is highlighted. The position of the KilA-N (-like) domain (BstA^BTP1^ residues 32–147) is indicated by a gray box. Heatmaps on the left of the alignment indicate the prophage and plasmid association of each homolog (lanes 1 and 2) and the taxonomic group each homolog derives from (lane 3). Prophage association was split into high and low confidence based on gene co-occurrence criteria (STAR Methods).

Having established that BstA functions as a prophage-encoded phage-defense system, we sought to further characterize the evolutionary conservation of this protein. We identified BstA homologs in the genomes of diverse Gram-negative bacteria (Table S1) and compiled a dataset of 72 homologs representative of phylogenetic diversity. The majority (79%) of BstA homologs co-occurred with phage genes and were designated as putatively prophage associated (Figure 2A). No known phage-associated genes were found in the vicinity of 21% (15 of 72) of BstA homologs, which were defined as putatively prophage independent. A small subset of BstA homologs were plasmid encoded (Figure 2A). Strikingly, in many cases, BstA homologs were located downstream of putative prophage repressor proteins, mirroring the genetic architecture of BstA^BTP1^ (Figure 2B). We conclude that the BstA protein is highly associated with prophages of Gram-negative bacteria.

While the BstA protein does not exhibit sequence homology to any functionally characterized proteins, remote homology detection methods revealed a KilA-N (-like) domain in the N-terminal region (residues 32–147 of BstA^BTP1^) (Figure 2C). Although poorly characterized, the KilA-N domain is found in proteins from phages and eukaryotic DNA viruses and contains the helix-turn-helix motif characteristic of DNA-binding proteins (Iyer et al., 2002; Medina et al., 2019).

Certain residues in the BstA protein are highly conserved among homologs from diverse members of the alpha, beta, and gamma proteobacteria (Figures 2C and S2A). A small number of BstA homologs only exhibited homology to the N-terminal, KilA-N (-like) domain. A second small group of homologs were only homologous to the C-terminal region of BstA (shown at the bottom of the alignment in Figure 2C). Such bipartite protein homology suggests that the BstA protein is composed of two functional domains. This conclusion is independently supported by evolutionary covariance analysis (Figure S2B), where the clear depletion of predicted residue contacts between the ranges 1 to ∼155 and ∼156–307 of BstA^BTP1^ suggests that there is a domain boundary (Rigden, 2002) around position 155, with the two folded domains making few contacts.

**Figure 3:**
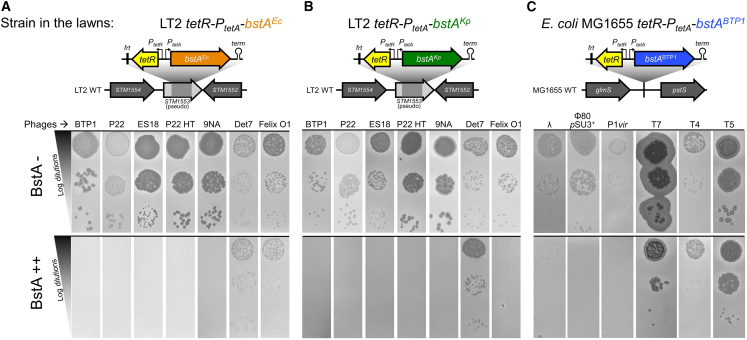
BstA homologs from *Salmonella*, *E. coli*, and *K. pneumoniae* confer phage defense (A and B) Heterologous expression of *bstA* homologs from (A) *E. coli* NCTC10963 (*bstA*^*Ec*^) and (B) *K. pneumoniae* Kp52.145 (*bstA*^*Kp*^) in *Salmonella* strain LT2 confers phage defense at similar levels to *bstA*^*BTP1*^ but shows additional activity against phage BTP1. (C) BstA^BTP1^ confers defense against coliphages in *E. coli* MG1655. Plaque assays were carried out with the indicated phages applied on mock-induced (BstA−) or AHT-induced (BstA++) lawns of LT2 *tetR-P*_*tetA*_*-bstA*^*Ec*^ (JH4408), LT2 *tetR-P*_*tetA*_*-bstA*^*Kp*^ (JH4404), or MG1655 *tetR-P*_*tetA*_*-bstA*^*BTP1*^ (JH4410). The genetic context for the *tetR*-*P*_*tetA*_-*bstA* insertions within the *SM1553* pseudogene of LT2 or in the *glmS-pstS* intergenic region of MG1655 is depicted above.

We selected two diverse BstA homologs from *Klebsiella pneumoniae* (BstA^Kp^, 48.4% amino acid identity to BstA^BTP1^) and *E. coli* (BstA^Ec^, 41.7% amino acid identity) to investigate the phage-resistance function of the larger BstA protein family (the native genetic context of these homologs is illustrated in Figure 2B, and their identity to BstA^BTP1^ is highlighted in the alignment in Figure 2C). We engineered inducible expression systems mirroring the expression construct previously validated for BstA^BTP1^ (Figures 3A, 3B, and S1C). The expression of BstA^Kp^ and BstA^Ec^ in *S.* Typhimurium LT2 conferred resistance to *Salmonella* phages at a similar level to BstA^BTP1^, despite these BstA homologs only sharing around 40% identity at the amino acid level (Figures 3A, 3B, and S1E). Unlike BstA^BTP1^, BstA^Kp^ and BstA^Ec^ prevented the replication of phage BTP1 (which encodes *bstA*^*BTP1*^).

Finally, we tested the function of BstA against well-characterized coliphages. Heterologous expression of BstA^BTP1^ in *E. coli* strain MG1655 conferred resistance to phage λ, ϕ80, P1, and T7 but did not affect phages T4 and T5 (Figure 3C). Surprisingly, we found that BstA^Ec^ was slightly less active against coliphages than BstA^BTP1^ (Figures 3C and S1E). Replication in liquid culture was a more reliable and reproducible measure of phage susceptibility than plaque assay and frequently revealed stronger resistance phenotypes than by plaque assay (Figures S1F and S1G).

We conclude that BstA represents a family of phage-resistance proteins associated with the prophages of diverse Gram-negative bacteria.

### BstA mediates effective population-level phage defense through abortive infection

**Figure 4:**
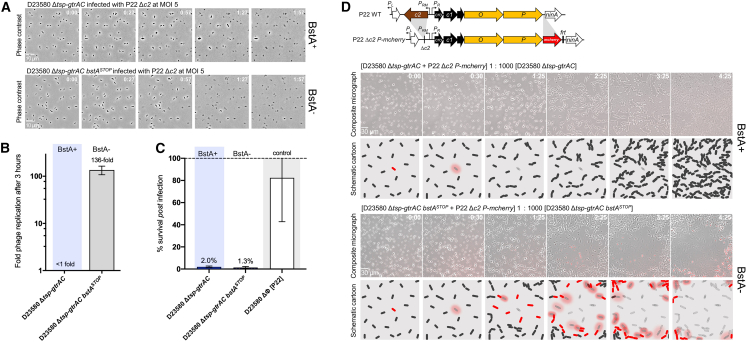
BstA mediates population-level phage defense through abortive infection BstA protein does not protect individual cells from phage infection (A) Cells natively expressing BstA (D23580 Δ*tsp-gtrAC*, JH4287) or possessing a mutated BstA locus (D23580 Δ*tsp-gtrAC bstA*^*STOP*^, SNW431) were infected with the obligately virulent P22-derivate phage, P22 Δ*c2*, at an MOI of 5 to increase the likelihood of infecting all cells. Infected cells were imaged on agarose pads and the images represent a time series. Regardless of BstA function, almost all cells were observed to lyse (indicated by loss of defined cell shape and phase contrast). Videos of the time series are presented in Video S1. A scale bar representing 10 μm is shown in the first image for each series. (B) A phage replication assay showed that P22 Δ*c2* phage failed to replicate after 3-h growth on the BstA+ strain (D23580 Δ*tsp-gtrAC*) but replicated ∼136-fold when BstA was inactivated (D23580 Δ*tsp-gtrAC bstA*^*STOP*^). (C) Survival assay of the same strains after infection by phage P22 Δ*c2*, at an MOI of 5. Consistent with the microscopy data in (A), BstA function did not affect cell survival from phage infection. D23580 ΔΦ [P22] (SSO-128), a phage P22 lysogen (and therefore natively resistant), was used as a negative control. Data in (B) and (C) are presented as the mean of biological triplicates ± SD. (D) A fluorescent reporter module for phage replication was added to P22 Δ*c2* (P22 Δ*c2 P-mCherry*) so that phage replication yielded red fluorescence (*mCherry* was inserted downstream of the replicative genes of the phage). A similar experiment to (A) was conducted, but P22 Δ*c2 P-mCherry* infected cells were mixed 1:1,000 with uninfected cells. In the BstA+ cells, primary infected cells lysed but did not stimulate secondary infections of neighboring cells, and they eventually formed a confluent lawn. In BstA cells, primary lysis events caused secondary infections (neighboring cells showing red fluorescence and subsequent lysis), causing an epidemic of phage infection reminiscent of plaque formation. Cartoons schematize the outcomes of these experiments in the two strain backgrounds. Representative micrographs of each time series are shown. Videos of the time series are presented in Video S2. All experiments were carried out in liquid or solid M9 Glu^+^ media. A scale bar representing 30 μm is shown in the first image for each series of micrographs.

Phage-resistance systems operate via diverse functional mechanisms (Hampton et al., 2020; Rostøl and Marraffini, 2019). We used microscopy to dissect BstA-mediated phage resistance. Virulent P22 phages (P22 Δ*c2*) were used to infect *Salmonella* cells with and without native BstA^BTP1^, at high multiplicity of infection (MOI) to ensure that most cells were infected. We were surprised to observe that independent of BstA^BTP1^, all cells lysed within the time course of 3 h (Figure 4A; Video S1), and BstA^BTP1^ did not appear to confer any direct protection from phage infection at the level of individual infected cells. We conducted the same experiment in liquid culture, measuring phage replication and the fraction of surviving cells post-phage infection. In cells possessing functional BstA (D23580 Δ*tsp-gtrAC*), phage P22 Δ*c2* completely failed to replicate (Figure 4B). In the absence of BstA function (D23580 Δ*tsp-gtrAC bstA*^*STOP*^), the phage replicated >100-fold. However, despite preventing the replication of phage P22, BstA^BTP1^ had no effect on cell survival: independent of BstA^BTP1^ function, only 1%–2% of cells survived following P22 infection (Figure 4C). We hypothesized that BstA does not protect single cells and instead mediates phage defense at the population level by sacrificing the life of the infected cell.

Video S1. BstA expression does not prevent exogenously infected cells from phage lysis, related to Figure 4 Cells natively expressing BstA (D23580 *Δtsp-gtrAC*, JH4287) or possessing a mutated BstA locus (D23580 Δ*tsp-gtrAC bstA*^*STOP*^, SNW431) were infected with the obligately virulent P22-derivate phage, P22 Δ*c2*, at an MOI of 5 (to increase the likelihood of infecting all cells). Infected cells were imaged every 3 minutes on agarose pads. Regardless of BstA activity, almost all cells were observed to lyse (indicated by loss of defined cell shape and phase contrast).

To investigate the BstA-protein-mediated population-level phage defense, we conducted a second microscopy experiment, wherein approximately only 1 in every 1,000 cells was infected with phage P22. Unlike culture in liquid media, our microscopy setup involved immobilization of cells on agarose pads, which allows only local movement of phage particles. The spread of infection was tracked as primary infected cells lysed and produced secondary infections in neighboring cells. To visualize these phage epidemics, we used a reporter phage engineered to encode the red fluorescent protein mCherry within the early lytic operon (P22 Δ*c2 P-mCherry*); the fluorescence signal indicated phage replication (Figure 4D).

In the population lacking functional BstA^BTP1^ (D23580 Δ*tsp-gtrAC bstA*^*STOP*^), primary infected cells lysed after around 30 min (Figure 4D; Video S2). Subsequently, the red fluorescence signal was observed in neighboring cells, revealing secondary infection, followed by cell lysis, a cycle that repeated until all cells in the radius of the primary infected cell had lysed, reminiscent of plaque formation (Figure 4D). The impact of the epidemic of phage infection upon bacterial cells lacking BstA^BTP1^ can be visualized in Video S2.

Video S2. BstA mediates population-level phage defense by suppressing phage epidemics by abortive infection, related to Figures 4 and S3 Cells natively expressing BstA (D23580 Δ*tsp-gtrAC*, JH4287) or possessing a mutated BstA locus (D23580 Δ*tsp-gtrAC bstA*^*STOP*^, SNW431) were infected with the obligately virulent P22-derivate phage, P22 Δ*c2* P-mcherry, at an MOI of 5 (to increase the likelihood of infecting all cells). Due to the mCherry insertion, red fluorescence corresponds to phage replication. Infected cells were mixed 1:1,000 with uninfected cells. Cells mixtures were imaged every 5 min on agarose pads for 6 h. In the BstA+ cells, primary infected cells lysed but did not stimulate secondary infections of neighboring cells, and they eventually formed a confluent lawn. In BstA cells, primary lysis events caused secondary infections (neighboring cells showing red fluorescence and subsequent lysis), causing an epidemic of phage infection reminiscent of plaque formation.

In contrast, in the D23580 Δ*tsp-gtrAC* population, no secondary infections were observed in the neighboring cells following the lysis of the primary infected cells. Instead, cells continued to divide normally, eventually forming a confluent lawn (Figure 4D; Video S2). The lack of subsequent rounds of infection after the primary cell lysis events shows that few or no infectious phage particles were generated.

Taken together, these experiments demonstrate that the BstA protein inhibits successful phage replication, but it does not prevent the death of the infected cell. Therefore, BstA provides phage defense at the population level and prevents the spread of phage epidemics. Accordingly, we propose that BstA is an abortive infection system.

### BstA protein responds dynamically to phage infection and colocalizes with phage DNA

To explore the molecular activity of BstA during phage infection, we first constructed a functional translational fusion of the BstA^BTP1^ protein to superfolder green fluorescent protein (_sf_GFP) (Figure S3A). We then used time-lapse fluorescence microscopy to observe the dynamics of the BstA protein inside individual cells during infection with two BstA-sensitive phages, P22 and 9NA. In the absence of phage infection, the BstA protein was distributed diffusely within the cytoplasm of the cells, suggesting no particular subcellular localization (Figures S3B and S3C; Video S3). However, approximately 20 min after infection with phages P22 and 9NA, we consistently observed BstA protein aggregating into discrete foci toward the center of infected cells (Figure S3C; Video S3). Cell lysis occurred approximately 40 min after the formation of BstA foci.

Video S3. BstA proteins form dynamic foci in response to phage infection, related to Figures 4 and S3 Cells natively expressing BstA translationally fused to _sf_GFP (D23580 *bstA-*_*sf*_*gfp*, SNW403) were grown in a microfluidic growth chamber and imaged every 1.5 min. Fluorescently labeled phages P22 HT (left) or 9NA (right) were then added to the cells and can be seen adsorbing to cells as red fluorescent puncta. For purposes of comparison, timestamps are synchronized to the point at which phage are first observed. Typically, around 20 min after initial observation of phage infection, BstA proteins formed discrete and dynamic foci within the. Cells then proceeded to lyse.

We speculated that the dynamic establishment of foci by BstA in response to phage infection was likely to reflect the mechanistic activity of the protein. We noticed that the foci dynamics of BstA proteins during phage infection resembled phage replisomes (Cenens et al., 2013; Trinh et al., 2017). Therefore, we speculated that the focus of the BstA protein in phage-infected cells might correspond to the replicating phage DNA. To test this hypothesis, we used a ParB-*parS* system to track the subcellular localization of phage DNA relative to BstA protein. We inserted a *parS* site into the P22 phage chromosome and expressed a ParB-mCherry fusion protein inside cells already expressing BstA-_sf_GFP_._ ParB protein oligomerizes onto DNA at *parS* sites, labeling *parS*-tagged DNA with ParB-mCherry foci. We conducted a microfluidic infection experiment to colocate BstA foci and infecting P22 phage DNA and observed that the position of ParB-mCherry foci (corresponding to phage P22 DNA) overlapped with foci formed by BstA-_sf_GFP (Figure S3D; Video S4). Therefore, the microscopy data suggest that BstA protein interacts with the replicating DNA of infecting phages. Consistent with the other microscopy data (Figures 4A and S3C), cells proceeded to lyse after the formation of BstA/ParB-mCherry foci. We note that the strain used in this experiment (SVO251; Table S2) is cured of all prophages, ruling out the possibility that cell lysis is caused by native prophage induction.

Video S4. BstA protein foci correspond to sites of infecting phage DNA, related to Figures 4, S3, and 6 Cells heterologously expressing BstA translationally fused to _sf_GFP, and ParB translationally fused to mCherry (D23580 ΔΦ ΔpSLT-BT ΔpBT1 *STMMW_15481*::[*P*_*tetA*_-*bstA*^*BTP1*^*-*_*sf*_*gfp-frt*] pAW61, SVO251) were grown in a microfluidic growth chamber and imaged every 2 min.
Phage P22 Δ*pid*::(*parS-aph*) were then added to the cells. ParB protein oligomerizes onto DNA at *parS* sites; therefore, *parS*-tagged DNA is indicated by ParB-mCherry foci (red). Translocation of infecting phage DNA into cells is indicated by the formation of red foci within the cells, which is rapidly followed by formation of green, BstA foci. The green and red foci appear to physically overlap. Infected cells proceed to lyse. The same time series is presented as separate channels (phase contrast, GFP, and mCherry) and a composite merge.

In summary, our data are consistent with a model that involves the movement of BstA protein to sites of phage DNA replication inside infected cells, followed by prevention of phage replication.

### BstA phage-resistance systems contain anti-BstA elements (*aba*) that suppress the activity of BstA

**Figure 5:**
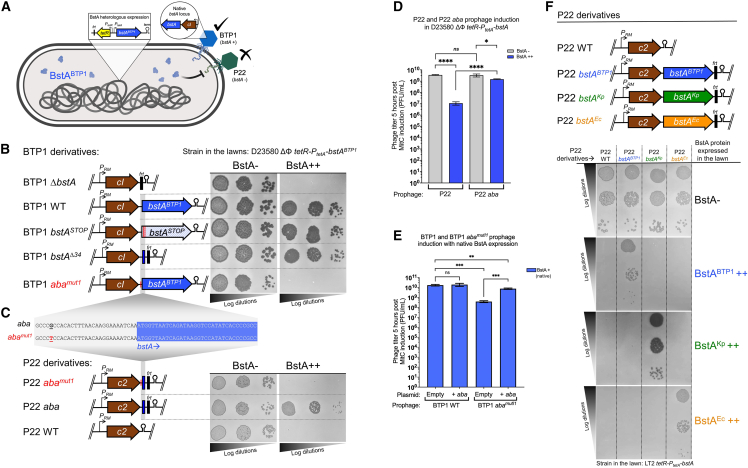
BstA systems include cognate self-immunity elements, *aba*, which are required for successful prophage induction (A) Cartoon summarizing the data from Figure 1E. The BTP1 phage, which encodes the *bstA* locus, is not affected by heterologous expression of BstA^BTP1^, while the replication of phage P22 is inhibited. <br>(B) Schematic of the BTP1-derived phages used and the corresponding effect on sensitivity to BstA^BTP1^ expression (plaque assay). (C) Schematic of the P22-derived phages used. In all cases, introduced sequences (*bstA* homologs or fragments) were inserted downstream of the *c2* repressor gene of P22 and are linked to the *frt* sequence. Hairpins represent Rho-independent terminators. Insensitivity of the BTP1 phage to BstA^BTP1^ is dependent on the *bstA* locus on the phage chromosome. However, only the first 34 bp of the *bstA* gene are required, along with 29 bp upstream (in total a 63-bp sequence termed *aba*, for anti-BstA). The *aba* sequence (native in BTP1 or engineered into P22) counteracts the BstA-driven phage resistance. The G→T mutation (*aba*^*mu1*^) causes loss of *aba* function and suppresses the anti-*bstA* interference. (D) BstA represses P22 prophage induction in the absence of *aba*. P22 induction was measured in D23580 ΔΦ *tetR-P*_*tetA*_*-bstA*^*BTP1*^ lysogenized with prophages P22 WT or P22 *aba* (strain SNW583 and SNW585, respectively). The induced phage titer was measured 5 h post induction with Mitomycin C (MitC). (E) Endogenous BstA represses BTP1 prophage induction in the presence of the *aba*^*mut1*^ mutation, but replication can be rescued by supply of a functional *aba* in *trans*. Prophage induction was measured in strain D23580 ΔΦ ΔTn*21* (Ap^S^) lysogenized with BTP1 WT (*aba*^*WT*^) or BTP1 *aba*^*mut1*^ (strain SNW597 and SNW598, respectively). Lysogens were transformed with pUC18 (vector) or pUC18-*aba* (pNAW203, +*aba*) and prophage induction was measured 5 h post induction with Mitomycin C. Data in (D) and (E) are presented as the mean of biological triplicates ± SD. Groups were compared using unpaired two-tailed Student’s t test. ∗∗∗∗p < 0.0001, ∗∗∗p = 0.0001–0.001, ∗∗p = 0.001–0.01, ∗p = 0.01–0.05; ns, p ≥ 0.05. (F) Each *bstA* locus encodes a homolog-specific anti-BstA element (*aba*) that suppresses BstA-mediated phage defense. Transfer of each *bstA* locus to phage P22 only confers immunity against the cognate BstA protein. Plaque assays were carried out with the indicated phages, applied on mock-induced (BstA−) or AHT-induced (BstA++) lawns of the indicated strain: SNW576 for (B) and (C) and strains JH4400 (BstA^BTP1^++), JH4404 (BstA^Kp^++), or JH4408 (BstA^Ec^ ++) for (F).

When characterizing the sensitivity of different phages to the activity of BstA^BTP1^ (Figure 1E), we observed that phage BTP1 (which itself encodes the *bstA*^*BTP1*^ gene) was not affected by heterologous expression of BstA^BTP1^ (schematized in Figure 5A). We hypothesized that BTP1 carried an anti-BstA determinant, which is as follows: a self-immunity factor that allows phage BTP1 to replicate without being targeted by its own abortive infection protein. Consistent with this hypothesis, phage BTP1 became sensitive to BstA^BTP1^ expression when the *bstA* coding sequence was deleted (BTP1 Δ*bstA*). (Figure 5B). The self-immunity function of the *bstA* locus was not affected by the introduction of the double stop codon mutation into the beginning of the coding sequence (as described in Figure 1C), indicating that self-immunity is not mediated by the BstA protein itself but by an alternative genetic element encoded within the *bstA* locus (Figure 5B). Here, and for the duration of this report, we define the *bstA* “locus” as the region, including the *bstA* coding sequence and its 5′ upstream sequence.

To identify the genetic basis of BstA self-immunity, we constructed a series of BTP1 mutant phages, carrying truncations of different lengths from the 3′ end of the *bstA* locus (Figure S4A) and screened these phages for the ability to replicate in the presence of BstA^BTP1^ expression. Self-immunity (i.e., insensitivity to BstA^BTP1^ expression) was preserved in all mutant phages except the mutant with the longest *bstA* truncation (BTP1 *bstA*^*Δ24*^) in which just the first 24 bp of the *bstA* reading frame were intact (Figure S4A). A similar truncation mutant containing just the first 34 bp of *bstA* (BTP1 *bstA*^*Δ34*^) retained immunity to BstA, suggesting that the first 34 bp of the *bstA* gene are essential for the activity of the anti-BstA determinant. The transfer of *bstA*^*Δ34*^ (the first 34 bp of *bstA*, along with the upstream sequence) to phage P22 (P22 *bstA*^*Δ34I*^) conferred BstA immunity (Figure S4B). To identify the minimal sequence required for BstA self-immunity, we further constructed P22 *bstA*^*Δ34I*^-derived phages, successively truncating the transferred sequence from the 5′ end (P22 *bstA*^*Δ34I*^-P22 *bstA*^*Δ34V*^, Figure S4B). We discovered that a 63 bp sequence (GCCCGCCACACTTTAACAAGGAAAATCAAATGGTTAATCAGATAAGGTCCATATCACCCCGCC) spanning 29 bp of the upstream region and the first 34 bp of the *bstA* coding sequence (start codon underlined) was necessary and sufficient to confer the self-immunity (Figure 5C). We designated this element “*aba*,” for anti-BstA. Supplying the 63 bp *aba* sequence on the high-copy-number pUC18 plasmid (pUC18-*aba*) rescued P22 phage replication in the presence of BstA protein, demonstrating that the self-immunity effect of *aba* is retained in *trans* (when *aba* is not carried by the targeted phage but is supplied on another replicative element) (Figure S5A). The intracellular localization of the BstA protein following phage infection was unaffected by the presence of the pUC18-*aba* plasmid (Figure S5B).

### The *aba* element appears to be DNA based

In the native BTP1 prophage, the *aba* sequence overlaps the start of the *bstA* gene, preventing mutational disruption of the *aba* element without modification of the BstA protein sequence. Therefore, we used the plasmid *trans*-complementation system (wherein the BstA protein and the *aba* sequence are independently encoded) to probe the function of the *aba* sequence (Figure S5A). A notable feature of the *aba* sequence is the presence of a direct “CCCGCC” repeat at the terminal ends, which we hypothesized was functionally important. Single-nucleotide exchange of the CCCGCC→CCCTCC in the first and second repeat (*aba*^*mut1*^ and *aba*^*mut2*^, respectively) abolished the self-immunity function of the *aba* element, both when located on a phage (Figure 5B) and from a plasmid in *trans* (Figure S5C), showing that the *aba* terminal direct repeats are required for *aba* function. Plasmid-borne expression of BstA efficiently suppressed plaque formation of P22 and BTP1 phages lacking a functional *aba* sequence (P22 WT, BTP1 Δ*bstA*, or BTP1 *aba*^*mut1*^) but had no effect on BTP1 WT, which natively encodes *aba* (Figure S5D).

The *aba* plasmid *trans*-complementation system additionally allowed us to interrogate the genetic nature of the *aba* element, which we hypothesized was either DNA, RNA, or peptide based. Although three short open reading frames exist within the *aba* sequence, nonsynonymous mutation of the reading frames did not ablate *aba* function (Figure S6A), suggesting the *aba*-driven immunity is not mediated by a small peptide.

Second, we investigated whether the *aba* element is DNA or RNA based by assessing whether transcription of *aba* is necessary for suppression of BstA. The *aba* sequence was cloned into the high-copy pUC18 vector with no promoter and flanked by terminators to abrogate transcription (Figure S6B). This created a scenario with high-copy *aba* DNA and minimal *aba* transcription. In parallel, we inserted the *aba* sequence into the *Salmonella* chromosome downstream of the arabinose-inducible *P*_*BAD*_ promoter (D23580 ΔΦ *tetR*-*bstA*^*BTP1*^ *P*_*BAD*_*-aba-gfp*; Figure S6B). In this scenario, *aba* exists as a single copy of DNA but is highly transcribed. In both plasmid and chromosomal constructs, a *gfp* gene was transcriptionally fused to the *aba* sequence to report the level of transcription. Our chromosomal *P*_*BAD*_*-aba-gfp* construct generated a high level of green fluorescence in our assay conditions, whereas fluorescence was barely detectable for our plasmid-based *aba* constructs (Figure S6B), demonstrating that much more *aba* RNA is transcribed from the single-copy chromosomal construct than the high-copy plasmid construct. We assessed the activity of BstA in both scenarios by challenging the cells against phages P22 and 9NA. *aba* only functioned to suppress the activity of BstA (i.e., allow plaquing of P22 and 9NA) in the high-copy DNA, low-transcription scenario, suggesting that the *aba* element is DNA based. However, a single chromosomal copy of *aba* did not confer self-immunity (Figure S6B), suggesting that *aba* DNA can only suppress BstA when supplied on high-copy replicative elements. Further mutational disruption of the *aba* sequence revealed that the self-immunity function was sensitive to mutation at multiple sites in the 63 bp sequence (Figure S6C).

Collectively, our data suggest that *aba*-driven suppression of BstA is neither peptide nor transcript mediated, and supports a model where BstA suppression is mediated by *aba* DNA.

### The *aba* element prevents the *bstA*-encoding prophage from aborting its own lytic replication

Unlike most mechanistically characterized abortive infection systems, a unique feature of the BstA system is its frequent occurrence on prophages (Figure 2A). Prophages must be able to switch to lytic replication, or else the prophage state becomes an evolutionary dead end for the phage.

We hypothesized that the primary biological role of the *aba* element is to allow the endogenous *bstA*-encoding phage to escape BstA-mediated inhibition upon induction from the prophage state. To test this, we measured the level of induction of prophage P22 in the presence of heterologously expressed BstA^BTP1^ protein (Figure 5D). In the absence of BstA^BTP1^ expression, the P22 prophage generated a titer of ∼4 × 10^9^ PFU/mL after 5-h growth with an inducing agent (Mitomycin C, MitC). However, with BstA^BTP1^ expression, the MitC-induced titer of P22 dropped >300-fold to ∼1 × 10^7^ PFU/mL, showing that BstA inhibited P22 phage replication. The transfer of the *aba* sequence to prophage P22 (P22 *aba*) significantly increased the induced titer in the presence of BstA^BTP1^ to ∼1.5 × 10^9^, restoring it to the level seen in the absence of BstA and showing that the *aba* element rescues prophage induction via suppression of BstA.

Finally, we validated the importance of the *aba* element in the context of native BTP1 prophage induction. The presence of additional copies of the *aba* sequence in *trans* on the pUC18 plasmid did not affect the titer of BTP1 phage generated after 5-h growth with an inducing agent (MitC), suggesting that native levels of the BstA protein do not constrain BTP1 prophage induction in the presence of the native, functional *aba* element (Figure 5E). However, when the *aba*^*mut1*^ mutation (exchange of a single functionally important nucleotide in the terminal direct repeat) was introduced into the BTP1 prophage, the MitC-induced titer of phage BTP1 was reduced ∼40-fold in the presence of native BstA expression. This reduction was almost entirely rescued when the *aba* sequence was supplied in *trans* on the pUC18 plasmid, confirming that the *aba*^*mut1*^ mutation ablates the function of the *aba* element. When the *aba*^*mut1*^ mutation was introduced into the BTP1 prophage in the absence of native BstA protein expression (D23580 ΔΦ [BTP1 *aba*^*mut1*^ *bstA*^*STOP*^]) there was no effect on prophage induction (Figure S6D), confirming that the effect of the *aba*^*mut1*^ mutation is dependent on the presence of BstA.

These experiments demonstrate that a functional *aba* element is required for the *bstA*-encoding prophage to switch from a lysogenic to lytic lifestyle. In the absence of *aba*, the *bstA*-encoding prophage suffers replication inhibition by endogenous BstA protein (self-targeting), presumably by the same abortive infection mechanism that inhibits exogenous phage infection.

### Distinct BstA proteins are associated with cognate *aba* elements

Finally, we determined whether the *aba* sequence from *bstA*^*BTP1*^ could suppress the activity of variant BstA proteins of other bacteria. We challenged the P22 *bstA*^*BTP1*^ phage (immune to expression of BstA^BTP1^ due to the presence of *aba^BTP1^*) against expression of BstA^Ec^ or BstA^Kp^. The *bstA*^*BTP1*^ locus did not protect P22 from the variant BstA proteins, suggesting that the *aba* element from *bstA*^BTP1^ only confers immunity again BstA^BTP1^ and therefore that variant BstA proteins have cognate *aba* elements (Figure 5F). To test this hypothesis, we engineered P22 phages to encode either *bstA*^*Ec*^ or *bstA*^*Kp*^ loci (including the respective upstream sequence). Consistent with a cognate BstA-*aba* interaction, P22 *bstA*^*Ec*^ became specifically immune to expression of BstA^Ec^, and P22 *bstA*^*Kp*^ gained specific immunity to BstA^Kp^ expression (Figure 5F).

We conclude that while BstA proteins are broadly functionally interchangeable in terms of their phage-defense activity, each *bstA* locus contains a cognate *aba* element that is inactive against variant BstA proteins. The specificity of the *aba* self-immunity element means that phages encoding *bstA* variants are unable to bypass all BstA-mediated abortive infection, making *aba*-mediated suppression of BstA exclusive to the corresponding induced *bstA*-encoding prophage.

### BstA protein does not affect phage lysogenic development but inhibits DNA replication during lytic development

**Figure 6:**
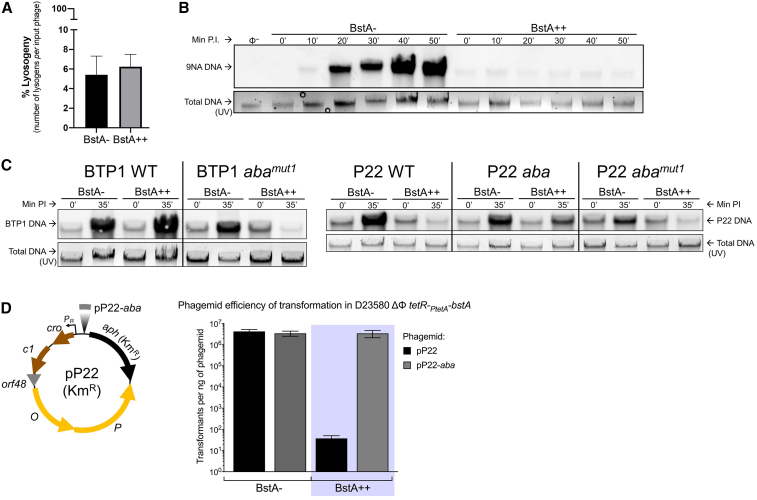
BstA protein does not affect phage lysogeny but inhibits phage DNA replication in the absence of *aba* (A) Frequency of lysogeny of the P22 Δ*pid*::*aph* phage in mock-induced (BstA−) or AHT-induced (BstA++) D23580 ΔΦ *tetR-P*_*tetA*_*-bstA*^*BTP1*^ (SNW576). Data are presented as the mean of biological triplicates ± SD. (B and C) DNA replication of 9NA phage (B), BTP1 and P22-derived phages (C) in the absence or presence of BstA expression. Phage DNA was detected by Southern blotting with total DNA extracted from mock-induced (BstA−) or AHT-induced (BstA++) host strain D23580 ΔΦ *tetR-P*_*tetA*_*-bstA*^*BTP1*^ (SNW576), infected by the indicated phage at MOI = 5. Before the transfer procedures, total stained DNA was visualized from gels under UV light and the resulting pictures served as loading control. Min P.I., minutes post infection. Non-infected SNW576 DNA was used as negative control to check the DNA probe specificity. (D) *aba* dramatically increases the transformation efficiency of P22-derived phagemids in BstA-expressing *Salmonella*. The Km^R^ phagemids pP22 (pNAW229) and pP22-aba (pNAW230) are schematized, and the efficiency of transformation for each phagemid was measured in mock- or AHT-induced competent bacteria of strain D23580 ΔΦ *tetR-P*_*tetA*_*-bstA*^*BTP1*^ (SNW576). Data are presented as the mean of biological triplicates ± SD.

To interrogate how the BstA protein interacts with infecting bacteriophages, we determined whether lysogenic phage development, where the infecting phage integrates into the genome of the bacterium, was affected by BstA expression. We used an antibiotic-tagged derivative of P22 (P22 Δ*pid*::*aph*) to determine the frequency of lysogeny with and without BstA expression. We found that the frequency of lysogeny was approximately 6% (Figure 6A) regardless of the presence of BstA, suggesting that BstA expression does not affect phage lysogenic development. This finding suggests that BstA activity is triggered by, or targets, an aspect of phage lytic replication not shared by lysogenic development. Further, it implies that BstA has no effect on the initial stages of phage infection that occur prior to lysogenic development, i.e., adsorption and DNA translocation.

The sequence-based analysis of BstA protein homologs suggested that the N-terminal domain may bind DNA (Figure 2C), and fluorescence microscopy showed BstA protein colocalizing with phage DNA (Figure S3D). The replication of DNA is crucial for phage morphogenesis, as a new copy of the phage chromosome is required for packaging into the capsid of each new virion. To test whether BstA protein inhibits phage DNA replication during lytic development in a manner that can be suppressed by *aba*, we conducted Southern blot experiments to monitor levels of phage DNA during infection. Using our prophage-negative, inducible BstA-expression strain (D23580 ΔΦ *tetR-P*_*tetA*_*-bstA*^*BTP1*^), we first tested the replication of the BstA-sensitive virulent phage, 9NA. In the absence of BstA expression, the level of phage 9NA DNA gradually increased over a 50-min infection time course, reflecting successful phage replication (Figure 6B). However, no accumulation of phage 9NA DNA was observed in the presence of BstA^BTP1^, suggesting that BstA protein strongly inhibited the replication of phage DNA.

Consistent with the self-immunity function of *aba*, BTP1 phage DNA replication was not affected by the expression of BstA^BTP1^, unless the *aba* element was nonfunctional (BTP1 *aba*^*mut1*^) (Figure 6C). Likewise, successful replication of phage P22 DNA in the presence of BstA^BTP1^ only occurred when the phage possessed a functional *aba* element (Figure 5B).

To confirm that BstA protein inhibits DNA replication, we constructed small phage-derived plasmids (“phagemids”) based on the phage P22 replication module (pP22) (Figure 6D) and a P22 phagemid that included the 63 bp *aba* sequence (pP22-*aba*).

*Salmonella* cells were transformed with the phagemids in the presence or absence of BstA^BTP1^ protein expression. In the absence of BstA, the stable replication of both P22 phagemids in *Salmonella* cells generated >10^6^ transformants/ng phagemid. However, the expression of BstA^BTP1^ reduced the transformation efficiency of pP22 (lacking the *aba* sequence) to around 10 transformants/ng. The addition of the *aba* sequence to the phagemid (pP22-*aba*) restored the transformation efficiency of the phagemid in the presence of BstA to BstA-negative levels (Figure 6D).

We conclude that phage DNA replication is strongly suppressed by BstA, but replication can be rescued by the *aba* element, presumably by suppression of BstA protein activity. As replicated phage DNA is an essential substrate for packaging into phage capsids, the inhibition of DNA replication is likely to prevent the production of infectious progeny phages, consistent with the observation that infectious phages are not released from BstA-expressing cells following cell lysis (Figure 4). We propose that BstA protein mediates abortive infection by suppressing phage DNA replication, a process that can be circumvented by the native prophage carrying the *aba* self-immunity element.

## Discussion

Here, we have discovered a family of prophage-encoded abortive infection proteins (BstA), which efficiently defend bacterial populations from phage epidemics. The BstA protein is constitutively expressed inside cells that carry the prophage and provides effective population-level phage defense through abortive infection, inhibiting phage replication at the cost of the viability of individual infected cells. Possession of such innate phage-defense systems by active prophages imposes the following challenge: the prophage must avoid self-targeting by its own defense system when switching to lytic replication.

The BstA system solves this problem with the *aba* element (anti-BstA), a co-encoded short DNA sequence that specifically suppresses the activity of BstA protein upon prophage induction, giving the induced prophage self-immunity against endogenous BstA protein. Theoretically, such a system might leave BstA-expressing cells vulnerable to infection by other BstA-encoding phages, which could use their own *aba* elements to bypass native BstA. This problem is avoided by cognate BstA-*aba* pairs, as each BstA protein is suppressed only by the cognate, co-encoded *aba* element, ensuring that BstA suppression is specific to the native BstA-encoding prophage.

Although we present a high-level overview of the BstA phage-defense system and the corresponding anti-BstA-*aba* element, we are left with two major questions regarding the activity of the BstA protein. First, what are the phage determinants for BstA sensitivity? Although BstA was active against approximately 50% of the phages tested, we did not detect similarities between BstA-targeted and non-targeted phages that could reflect the molecular determinants of sensitivity. It is possible that rather than responding to a physical phage stimulus, such as phage DNA or protein, BstA protein responds to a cellular stimulus produced by the infection of specific types of phages, for example, the recruitment of DNA replication machinery.

Second, what is the molecular mechanism by which BstA protein inhibits phage DNA replication? Our data suggest that phage DNA does not replicate in the presence of BstA. Although numerous Abi systems in *Lactococcus* have been proposed to interfere with phage DNA replicative functions (Chopin et al., 2005), the molecular mechanisms have not been well characterized. The existence of a putative DNA-binding domain in BstA proteins and the microscopic observation of BstA colocalization with phage DNA make it tempting to speculate that BstA interacts physically with phage DNA to prevent replication, for example, by occlusion of a replication initiation site.

Alongside the mechanistic details of the BstA protein that remain to be established, little is known about the interaction of BstA with the *aba* element. Our data show that *aba* interacts with BstA in DNA form, but the mechanism by which *aba* DNA suppresses the BstA protein is unclear. Our findings indicate that multiple copies of the *aba* element are required to suppress BstA protein in *trans*. However, copy number cannot be the only factor affecting *aba* functionality because a prophage is evidently able to suppress the BstA protein right from the initial stages of prophage induction, when *aba* is present as just a single copy on the chromosome. While we did not observe a loss of BstA focus formation during suppression with high-copy *aba* DNA, we are cautious to interpret this as evidence against a direct interaction between *aba* DNA and BstA protein. It remains possible that the phage-DNA colocalization behavior and abortive activity of BstA proteins are mechanistically uncoupled (indeed, BstA is predicted to contain two domains; Figure 2) or that reduction in focus formation is beyond the sensitivity of our microscopy methods. Further study of the BstA-*aba* system is required to resolve the precise molecular mechanisms by which BstA-encoding prophages, such as BTP1, achieve self-immunity.

We consistently observed that phage-infected cells that contained BstA protein underwent lysis, probably in the absence of infectious progeny phage release. However, we cannot be certain whether the BstA protein acts actively or passively to cause cell lysis. Abi systems have frequently been termed “altruistic suicide” systems, which mediate “programmed cell death” in response to phage infection (Abedon, 2012; Shub, 1994). While perhaps a useful conceptual analogy for the strictly population-level effect of Abi systems, this narrative implies that Abi systems actively cause cell death. Although this may often be the case, such as in the CBASS system (Cohen et al., 2019), Abi can also be achieved by simple disruption of the phage replication pathway. Because phage lysis is generally a temporally programed event that occurs independently of successful virion morphogenesis (Cahill and Young, 2019), phage-mediated cell lysis can occur in the absence of virion assembly. For example, many *Lactococcus* Abi systems target aspects of phage replication, such as AbiZ, which is thought to interact with phage holin proteins, to stimulate premature cell lysis before virion assembly is completed (Durmaz and Klaenhammer, 2007).

It is possible that the BstA protein simply inhibits viable phage particle formation, while allowing the phage lytic pathway to proceed unperturbed to cell lysis. However, inhibition of phage DNA replication would dramatically reduce substrates for transcription and translation of phage lysis gene products; yet, we did not observe a difference in the timing of cell lysis for phage-infected cells in the presence or absence of BstA during microscopy studies. The exact mechanism of cell lysis during BstA Abi activity will require further study.

An intriguing feature of the BstA phage-defense system is its tight association with prophages, and specifically, with the prophage repressor locus. Although we found homologs in diverse Gram-negative bacteria, the genetic architecture of the *bstA* locus (i.e., lying downstream of and presumably sharing the promoter of the prophage repressor) was strikingly conserved. The region between the repressor (*cI*) and *n* gene of lambdoid phages has previously been identified as a hotspot of mosaic diversity (Degnan et al., 2007). In fact, the corresponding site in phage Lambda harbors the *rexAB* genes, perhaps the most widely studied prophage-encoded abortive infection system (Snyder, 1995). Despite >60 years of research, the molecular mechanisms of RexAB activity are poorly understood. RexB is reported to be an ion channel, which triggers the loss of cell membrane potential upon activation by the intracellular sensor RexA (Labrie et al., 2010; Snyder, 1995). While not mechanistically comparable to BstA, perhaps the shared synteny of the BstA and RexAB abortive infection systems points to a functional significance of this genomic region, as the *cI* repressor gene is one of the most highly transcribed prophage promoters during lysogeny.

Although somewhat functionally analogous to toxin-antitoxin systems, to the best of our knowledge, no other examples of self-immunity mechanisms have been described within prophage-encoded abortive infection systems. However, some evidence supports the widespread existence of such mechanisms. For example, the activity of Lambda RexB protein can be suppressed by the overexpression of the *rexB* gene relative to *rexA*. It has been speculated, but not shown experimentally, that high levels of RexB might allow phage Lambda to replicate lytically in the presence of RexAB (Parma et al., 1992), i.e., giving the Lambda prophage self-immunity against its own Abi proteins.

In conclusion, the discovery of the BstA-*aba* system opens unexplored avenues of research into the mechanisms used by prophages to suppress their own phage-defense activities. We anticipate that similar strategies may be widespread and commonplace, perhaps existing within known prophage-encoded phage-defense systems. Given the huge mosaic diversity of temperate phages and high prevalence of uncharacterized accessory genes, the reservoir of prophage-encoded phage-defense and self-immunity systems is likely to be vast and largely unexplored.

## STAR★Methods

### Key resources table

**Table:** 

REAGENT or RESOURCE	SOURCE	IDENTIFIER
**Antibodies**		

Anti-Digoxigenin-AP, Fab fragments (Roche)	MilliporeSigma	Cat# 11093274910; RRID: AB_2734716

**Bacterial and virus strains**		

All the bacterial strains and bacteriophages are listed and described in Table S2	N/A	N/A

**Chemicals, peptides, and recombinant proteins**		

L-(+)-arabinose	Melford	Cat#A51000-100.0
Betaine	MilliporeSigma	Cat#B2629
Bacto Agar (BD)	Appleton Woods	Cat#MN663
Bacto Casamino Acids Technical (BD)	Appleton Woods	Cat#223110
EGTA	MilliporeSigma	Cat#E3889
M9 Salts 5X	MilliporeSigma	Cat#M6030
Maltose	MilliporeSigma	Cat#M5885
*m*-toluic acid	MilliporeSigma	Cat#T36609
Tryptone (BD)	Appleton Woods	Cat#MN649
Yeast Extract (BD)	Appleton Woods	Cat#DM832
Sodium Chloride	MilliporeSigma	Cat#S3014
Ampicillin Sodium	Melford	Cat#A40040-25.0
Anhydrotetracycline hydrochloride (AHT)	MilliporeSigma	Cat#37919
Chloramphenicol	MilliporeSigma	Cat#C0378
Gentamicin sulfate	Melford	Cat#G38000-25.0
Kanamycin monosulfate	Melford	Cat#K22000-25.0
Mitomycin C	MilliporeSigma	Cat#M0503
Tetracycline hydrochloride	MilliporeSigma	Cat#T7660
EcoRI	ThermoFisher Scientific	Cat#ER0271
BamHI	ThermoFisher Scientific	Cat#ER0051
DpnI	New England Biolabs	Cat#R0176S
KpnI-HF	New England Biolabs	Cat#R3142S
BsaI-HF	New England Biolabs	Cat#R3535S
SmaI	ThermoFisher Scientific	Cat#ER0661
SalI	ThermoFisher Scientific	Cat#ER0641
XbaI	New England Biolabs	Cat#R0145S
T4 Polynucleotide Kinase	ThermoFisher Scientific	Cat#EK0031
T4 DNA ligase	ThermoFisher Scientific	Cat#EL0014
CutSmart buffer	New England Biolabs	Cat#B7204S
Tango Buffer 10X	ThermoFisher Scientific	Cat#BY5
T4 DNA ligase Reaction Buffer	New England Biolabs	Cat#B0202S
Phusion High Fidelity DNA polymerase	New England Biolabs	Cat#M0530S
MyTaq Red PCR mix 2X	Bioline	Cat#BIO-25043
Taq DNA polymerase	Bioline	Cat#BIO-21105
NEBuilder HiFi DNA Assembly Cloning Kit	New England Biolabs	Cat#E5520S
DNase I	MilliporeSigma	Cat#DN25
RNase A	MilliporeSigma	Cat#R6513
Proteinase K	Bioline	Cat#BIO-37037
dNTP mix	Bioline	Cat#BIO-39025
Datp	Bioline	Cat#BIO-39036
Dgtp	Bioline	Cat#BIO-39037
dCTP	Bioline	Cat#BIO-39038
Dttp	Bioline	Cat#BIO-39039
DIG-11-dUTP, alkali-stable (Roche)	MilliporeSigma	Cat#11093088910
Midori Green DNA/RNA staining	Nippon Genetics	Cat#MG06
Electroporation cuvettes	Geneflow	Cat#E6-0060
Nylon membrane, positively charged (Roche)	MilliporeSigma	Cat#11417240001
Blocking Reagent (Roche)	MilliporeSigma	Cat#11096176001
DIG Easy Hyb Granules (Roche)	MilliporeSigma	Cat#11796895001

**Critical commercial assays**		

ISOLATE II Plasmid Mini Kit	Bioline	Cat#BIO-52057
ISOLATE II PCR and Gel Kit	Bioline	Cat#BIO-52060
Norgen Phage DNA Isolation Kit (46850)	GeneFlow	Cat#P4-0134
Quick-DNA Universal Kit	Zymo	Cat#D4069
Invitrogen Qubit dsDNA HS Assay Kit	ThermoFisher Scientific	Cat#Q32851
SYTOX Orange Nucleic Acid Stain – 5 mM in DMSO	ThermoFisher Scientific	Cat#S11368
Amicon Ultra-15 centrifugal filter units	MilliporeSigma	Cat#UFC910024

**Oligonucleotides**		

All the DNA oligonucleotides are listed in Table S2	N/A	N/A

**Recombinant DNA**		

All the plasmids are listed and described in Table S2	N/A	N/A

**Software and algorithms**		

GraphPad Prism 8.4.1	N/A	N/A
HMMER webserver	N/A	https://www.ebi.ac.uk/Tools/hmmer/
Prokka 1.13	N/A	N/A
HHPred webserver	N/A	https://toolkit.tuebingen.mpg.de/tools/hhpred
EMBOSS Needle webserver	N/A	https://www.ebi.ac.uk/Tools/psa/emboss_needle/

### Resource availability

#### Lead contact

Further information and requests for resources and reagents should be directed to and will be fulfilled by the lead contact, Siân Owen (sianvictoriaowen@gmail.com).

#### Materials availability

All unique bacterial, phage strains, and plasmids generated in this study are available from the lead contact without restriction.

### Experimental model and subject details

#### Bacteria and bacteriophages

The full list of bacterial strains used and constructed is available in Table S2. All the *Salmonella* strains were derived from the African *S.* Typhimurium ST313 strain D23580 (GenBank: FN424405.1) (Kingsley et al., 2009) or the model *S.* Typhimurium strain LT2 (GenBank: AE006468.2) (McClelland et al., 2001; Zinder and Lederberg, 1952). All the *Escherichia coli* strains constructed were derived from *E. coli* strain K-12 substrain MG1655 (GenBank: NC_000913.3) (Riley et al., 2006). The *bstA* homolog genes were cloned from *E. coli* NCTC10963 (GenBank: NZ_CAADJH010000002.1) or from *K. pneumoniae* Kp52.145 (GenBank: FO834906.1) (Bialek-Davenet et al., 2014). Bacteriophages (phages), including the temperate phages P22 (GenBank: NC_002371.2) (Pedulla et al., 2003) and BTP1 (GenBank: NC_042346.1) (Owen et al., 2017) and their derivatives, are described in Table S2. The genomic coordinates and gene identifiers indicated below refer to the GenBank accession numbers mentioned above.

### Method details

#### Growth conditions and transformation

All suppliers of chemical and reagents are specified in the key resources table. Unless stated otherwise, bacteria were grown at 37°C in autoclaved Lennox Broth (LB: 10 g/L Bacto Tryptone, 5 g/L Bacto Yeast Extract, 5 g/L NaCl) with aeration (shaking 220 rpm) or on LB agar plates, solidified with 1.5% Agar. The salt-free LBO media contained 10 g/L Bacto Tryptone, 5 g/L Bacto Yeast Extract. Pre-cultures were inoculated with isolated colonies from agar plates and grown to stationary phase (for at least 6 hours) in 5 mL LB in 30 mL universal glass tubes or in 50 mL plastic tubes (Greiner).

Cultures were typically prepared by diluting the pre-cultures (1:100) or (1:1000) in LB, and bacteria were grown in conical flasks containing 10% of their capacity of medium (*i.e.* 25 mL LB in a 250 mL conical flask) with aeration. For fluorescent microscopy experiments, bacteria were grown in M9 minimal medium (Sambrook and Russell, 2001) supplemented with 0.4% glucose and 0.1% Bacto Casamino Acids Technical (M9 Glu^+^).

When required, antibiotics were added to the media: 50 μg/mL kanamycin monosulfate (Km), 100 μg/mL Ampicillin sodium (Ap), 25 μg/mL tetracycline hydrochloride (Tc), 20 μg/mL gentamicin sulfate (Gm), 20 μg/mL chloramphenicol (Cm). Bacteria carrying inducible constructs with genes under the control of the *P*_*BAD*_ or *P*_*m*_ promoters were induced by adding 0.2 % (w/v) L-(+)-arabinose or 1 mM *m*-toluate, respectively. For the strains carrying *tetR-P*_*tetA*_ modules, *P*_*tetA*_ induction was triggered by adding 500 ng/mL of anhydrotetracycline hydrochloride (AHT, stock solubilized in methanol). For these constructs, the same volume of methanol was added to the non-induced cultures (mock treatment). Chemically-competent *E. coli* were prepared with RbCl-based solutions and were transformed by heat shock (Green and Rogers, 2014).

For the preparation of electro-competent cells, bacteria were grown in the salt-free medium LBO to an Optical Density at 600 nm (OD_600_) of 0.4-0.5. The bacteria were washed twice with cold sterile Milli-Q water (same volume as the culture volume) and were concentrated 100 times in cold 10% glycerol, prior to storage at -80°C. When ultra-competent *Salmonella* cells were required, the bacteria were grown in LBO at 45°C to OD_600_ 0.4-0.5, because growth at high temperature inactivates the *Salmonella* restriction systems (Edwards et al., 1999). Competent cells (10-50 μL) were mixed with 10-5000 ng of DNA in electroporation cuvettes (2 mm gap) and the reactions were electroporated (2.5 kV) using a MicroPulser electroporator (Bio-Rad). Bacteria were re-suspended in 0.5-1 mL LB and incubated for recovery at 37°C (30°C for temperature sensitive plasmids) with aeration, for at least one hour. Finally, the transformed bacteria were spread on selective LB agar plates and transformant colonies were obtained after at least 12 hours incubation at 30-37°C.

For assessment of strain growth kinetics with BstA expression, a FLUOstar Omega plate reader (BMG LABTECH) was used as follows: bacteria were inoculated at an initial OD_600_ of 0.01 (six replicates) in 200 μL of LB or LB + AHT in 96-well plates (Greiner). Bacteria were grown at 37°C with aeration (500 rpm, orbital shaking) and the OD_600_ was monitored every 15 min for 15 hours. Uninoculated LB medium was used as blank.

#### Cloning procedures

All the plasmids and DNA oligonucleotides (primers) are listed in Table S2. DNA manipulation and cloning procedures were carried out according to the enzyme and kit supplier recommendations and to standard procedures (Sambrook and Russell, 2001). DNA purity and concentration were measured with a DeNovix DS-11 FX spectrophotometer/fluorometer and using the Qubit dsDNA HS assay Kit.

For all the cloning procedures, Polymerase Chain Reactions (PCRs) were performed with the Phusion High Fidelity DNA polymerase, purified template DNA and primers in the presence of 3 % Dimethyl Sulfoxide and 1 M betaine, when required. Prior to Sanger sequencing of the constructs, PCR reactions were carried out directly from bacteria or phages with MyTaq Red Mix 2X. PCR fragments were analysed by electrophoresis, purified and finally sequenced with the appropriate primers (Lightrun service, Eurofins Genomics) (Table S2).

All the plasmids were constructed as detailed in the Table S2 and were verified by Sanger sequencing. Insertions of DNA fragments into plasmids were performed by digestion/ligation procedures, using restrictions enzymes and the T4 DNA ligase. In addition, PCR-driven restriction-free cloning techniques were used: overlap extension PCRs (Heckman and Pease, 2007) and plasmid assembly by PCR cloning (Van Den Ent and Löwe, 2006) were performed with chimeric primers, purified template DNA and Phusion DNA polymerase, as described previously (Owen et al., 2020). Cloning reactions were transformed by heat shock into *E. coli* Top10 (Invitrogen) or S17-1 λ*pir* (Simon et al., 1983). New template plasmids were constructed to insert fluorescent protein encoding genes into *Salmonella* or *E. coli* chromosomes, as reported previously (Gerlach et al., 2007). These plasmids carry the *ori*R6K γ origin of replication of pEMG, the *frt*-*aph*-*frt* (Km^R^) module of pKD4 linked to *gfp*^+^ (pNAW52), _*sf*_*gfp* (encoding for superfolder GFP, pNAW62) or *mcherry* (pNAW73), amplified respectively from plasmids pZEP09, pXG10-SF (Corcoran et al., 2012) and pFCcGi (Figueira et al., 2013). A similar template plasmid, carrying the *frt-aph-frt-tetR*-*P*_*tetA*_ module (pNAW55) was constructed and was used to insert the *tetR* repressor and the AHT-inducible promoter *P*_*tetA*_ upstream of genes of interest, as reported earlier (Schulte et al., 2019). For the construction of gentamicin resistant plasmids, the *aacC1* resistance gene was obtained from plasmid pME4510 (Rist and Kertesz, 1998).

The high copy number plasmid pUC18 was used to clone the different versions of the anti-*bstA* (*aba*) fragment: the *aba* fragments (*aba1-aba14* alleles) were amplified by PCR, digested with EcoRI and BamHI and ligated into the corresponding sites of pUC18. For cloning of the *aba* fragments fused to *gfp+* and flanked by terminators, 20nt overlapping DNA fragments were amplified with Q5 high fidelity polymerase, pooled and digested with DpnI prior to four piece isothermal assembly using the NEBuilder HiFi DNA Assembly Cloning Kit.

Phagemids based on the phage P22 replication module were constructed by EcoRI/KpnI digestion and ligation, as follows: the *P*_*R*_ promoter and the *cro-c1-orf48-O-P* genes of P22 (coordinates 31648-34683) were amplified and circularized by ligation with the *aph* Km^R^ cassette of pKD4 or with the *aba*-*aph* modules, amplified from strain SNW617. The ligation reactions were purified and electroporated into ultra-competent SNW555, a prophage-free and plasmid-free derivative of *S.* Typhimurium D23580. The resulting phagemids pNAW229 (pP22-*aph*), pNAW230 (pP22-*aba*-*aph*) were obtained after selection on Km medium.

Phage DNA was extracted from high titer lysates in LBO: nine volume of the phage lysates were mixed with one volume of 10 X DNase buffer (100 mM Tris-HCl, 25 mM MgCl_2_, pH 7.5) supplemented with RNase A (40 μg/mL final) and DNase I (400 μg/mL final). After 1 hour incubation at 37°C, DNase I was heat-inactivated at 75°C for 10 min and phage DNA was extracted from 500 μL of the nuclease-treated lysates with the Norgen Phage DNA Isolation after Proteinase K treatment, as specified by the manufacturer.

#### Genome editing techniques

Strain constructions are detailed in Table S2. For chromosomal insertions and deletions, *λ red* recombination was carried out with the arabinose-inducible plasmid pKD46 (for *E. coli*) or with the heat inducible plasmid pSIM5-*tet* (for *Salmonella*), both expressing the *λ red* genes. Bacteria were grown to exponential phase in LBO, according to the resistance and induction condition of the respective *λ red* plasmid (Datsenko and Wanner, 2000; Hammarlöf et al., 2018; Koskiniemi et al., 2011) and electro-competent cells were prepared as mentioned above. PCR fragments carrying a resistance cassette were constructed by overlap extension PCR or were directly obtained by PCR from the appropriate plasmid or strain. Electro-competent cells (40-50 μL) were transformed with 500-5000 ng of the PCR fragments and the recombinants were selected on selective LB agar plates.

Mutations or insertions linked to selective markers were transduced into *Salmonella* strains using the P22 HT *105/1 int-201* (P22 HT) transducing phage (Owen et al., 2017; Schmieger, 1972). For *E. coli*, the transducing phage P1 *vir* was used (Ikeda and Tomizawa, 1965; Tiruvadi Krishnan et al., 2015). Transductants were grown on selective LB agar plates supplemented with 10 mM EGTA. After two passages, clearance of the transducing phages was confirmed by diagnostic PCR using primer pairs NW_62/NW_63 for P22 HT or NW_392/NW_393 for P1 *vir* and by a passage on Green Agar medium (Maloy, 1990).To remove the antibiotic cassettes, flanked by FLP recognition target sites (*frt*), the FLP recombinase expressing plasmids pCP20, pCP20-TcR and pCP20-Gm were used, as previously reported (Cherepanov and Wackernagel, 1995; Doublet et al., 2008; Hammarlöf et al., 2018; Kintz et al., 2015). The inducible *tetR-P*_*tetA*_*-bstA* modules were constructed by fusing the *frt-aph-frt-tetR*-*P*_*tetA*_ module of pNAW55 to the *bstA* gene of D23580 (*bstA*^*BTP1*^, *STMMW_03531*), *E. coli* NCTC10963 (*bstA*^*Ec*^, *E4V89_RS07420*) or *K. pneumoniae* Kp52.145 (*bstA*^*Kp*^, *BN49_1470*). Each construct carries the native *bstA* ribosome binding site and Rho-independent terminator. The *tetR-P*_*tetA*_*-bstA* modules were inserted by *λ red* recombination into the *STM1553* pseudogene of *S.* Typhimurium LT2 (between coordinates 1629109-1629311), corresponding to *STMMW_15481* in D23580 (coordinates 1621832-3). Previously we have shown that the *STM1553* and *STMMW_15481* genes are not expressed at the transcriptional level (Canals et al., 2019).

In *E. coli* MG1655, the *bstA* modules were inserted into the *glmS-pstS* intergenic region (coordinates 3911773-4). To generate Ap and Cm sensitive D23580 strains, the pSLT-BT plasmid-encoded Tn*21*-like element, that carries the resistance genes (Kingsley et al., 2009), was replaced by the Km^R^ cassette of pDK4 by *λ red* recombination (deletion coordinates 34307 to 57061, GenBank: NC_013437.1). The resulting large single-copy plasmid pSLT-BT ΔTn*21*::*aph* was extracted (Heringa et al., 2007) and electroporated into the strains of interest. After selection on Km medium, the Ap and Cm sensitivity was confirmed and the Km^R^ cassette was flipped out using pCP20-Gm. For scarless genome editing, the pEMG plasmid-based allelic exchange system was used (Martínez-García and de Lorenzo, 2011). The pEMG derivative suicide plasmids were constructed as specified in Table S2 and were replicated in *E. coli* S17-1 *λpir*. Conjugation of the resulting plasmids into *Salmonella* and subsequent merodiploid resolution with plasmid pSW-2 were carried out as previously described (Canals et al., 2019; Owen et al., 2017). Key strains and phages (indicated in Table S2) used in this study were verified by whole-genome sequencing (Illumina) at MicrobesNG (Birmingham, UK).

#### Plasmid deletion in *S.* Typhimurium D23580

The pSLT-BT, pBT1, pBT2 and pBT3 plasmids (Kingsley et al., 2009) were cured from strain D23580, using the CRISPR-Cas9-based methodology (Lauritsen et al., 2017). A CRISPR-Cas9 Km resistant plasmid (pNAW136) was obtained by ligating the CRISPR-Cas9 module of plasmid pCas9 (Jiang et al., 2013) with the unstable origin of replication *ori*RK2, the *trfA* replication gene and the *aph* Km^R^ gene. Anti-plasmid protospacers (30 bp) were generated by the annealing of 5’-phosphorylated primer pairs that targeted the pSLT-BT, pBT1, pBT2 and pBT3 plasmids, designed according to the Marraffini Lab protocol (Jiang et al., 2013). The protospacers were ligated into *Bsa*I-digested pNAW136 with T4 DNA ligase and the resulting plasmids were checked by Sanger sequencing, using primer NW_658.

The resulting plasmids pNAW168 (anti-pSLT-BT) and pNAW169 (anti-pBT1), pNAW139 (anti-pBT2) and pNAW191 (anti-pBT3) were electroporated into D23580-derived strains and transformants were selected on Km plates. After two passages on Km, the loss of the pSLT-BT, pBT1, pBT2 or pBT3 plasmids was confirmed by diagnostic PCR. The absence of the unstable pNAW136-derived plasmids was confirmed by the Km sensitive phenotype of colonies after two passages on non-selective medium.

#### Phage stock preparation and plaque assays

All phage stocks were prepared in LB or LBO. For *Salmonella* phages, the prophage-free strain *S.* Typhimurium D23580 ΔΦ (JH3949) was used as host (Owen et al., 2017). Exponential phase cultures of D23580 ΔΦ were infected with ∼10^5^ Plaque Forming Units (PFU) and infected cultures were incubated for at least 3 hours at 37°C (with aeration). Phage lysates were spun down (4,000 X *g*, 15 min) and supernatants were filter-sterilized (0.22 μm, StarLab syringe filters). The resulting phage lysates were stored at 4°C in the presence 1% chloroform to prevent bacterial contamination.

Coliphage lysates were prepared similarly with *E. coli* MG1655 as host. When required, maltose (0.2%), CaCl_2_ (10 mM) and MgSO_4_ (10 mM) were added during the infection (λ, P1 *vir* and Φ80*p*SU3^+^). For Φ80-derived phages, the infection temperature was reduced to 30°C (Rotman et al., 2010).

Phage lysates were serial-diluted (decimal dilutions) with LB and virion enumeration was performed by double-layer overlay plaque assay (Kropinski et al., 2009), as follows. Bacterial lawns were prepared with stationary phase cultures of the reporter strains, diluted 40 times with warm Top Agar (0.5 % agar in LB, 50°C). The seeded Top Agar was poured on LB 1.5% agar bottom layer: 4 mL for 8.6 cm diameter petri dishes or 8 mL for 12 x 12 cm square plates.

When inducible *P*_*tetA*_ or *P*_*BAD*_ constructs were present in the reporter bacteria, 500 ng/mL of AHT or 0.2 % arabinose were added in the Top Agar. When required, antibiotics were added in the Top Agar layer. The bacterial lawns were incubated for 30 min at room temperature with the appropriate inducer, to allow solidification and the expression of the inducible genes. Finally, phage suspensions (5-20 μL) were applied on the Top Agar surface and pictures of the resulting plaques were taken with an ImageQuant LAS 4000 imager (GE Healthcare) after 16-20 hours incubation at 30 or 37°C.

#### Construction of P22 virulent phages

For the generation of obligately virulent P22 phages, a 633 bp in-frame deletion (coordinates 31028-31660) was introduced in the *c2* repressor gene by *λ red* recombination in a P22 lysogen as follows. Two fragments of ∼500 bp, flanking *c2*, were amplified with primers pairs NW_818 / NW_819 and NW_820 / NW_821. The two amplicons were fused by overlap extension PCR and 1000-3000 ng of the resulting Δ*c2* fragment were electroporated into P22 lysogens (in the prophage-free D23580 ΔΦ background) carrying the *λ red* recombination plasmid pSIM5-*tet*, as described above. The transformation reactions were re-suspended in 5 mL LB and incubated for 2 hours at 37°C with aeration. The culture supernatants were filter sterilized and serial-diluted to 10^-2^. Ten microliters of each dilution were mixed with 100 μL of a D23580 ΔΦ stationary phase culture and with 4 mL of warm Top Agar. The mixtures were poured on LB agar plates and the plates were incubated for ∼16 hours at 37°C. P22 Δ*c2* recombinants were identified by the clear morphology of their plaques, compared to the turbid plaques of WT P22. The Δ*c2* deletion was confirmed by PCR and Sanger sequencing with primers NW_406 and NW_805.

#### Use of the Δ*tsp-gtrAC* genetic background

Where possible, experiments were carried out with native BstA expression (from its natural locus within the BTP1 prophage), to best recapitulate the natural biological activity of the protein. However, as the *gtr* locus of phage BTP1 blocks attachment of many phages including P22 and BTP1, to achieve efficient phage infections we consistently used a strain background where the *gtr* locus has been inactivated (Δ*tsp-gtrAC*). The BTP1 prophage spontaneously induces to a titer of ∼10^9^ PFU/mL in liquid culture (Owen et al., 2017), and in the absence of *gtr* activity in surrounding cells, free BTP1 phages mediate cleavage of the O-antigen *via* the putative enzymatic activity of the tailspike protein (Kintz et al., 2015). Consequently, to avoid an unnatural, short LPS phenotype as a result of *gtr* inactivation in a BTP1 lysogen, we additionally inactivated the upstream gene encoding the BTP1 tailspike (*tsp*) (D23580 Δ*tsp-gtrAC*, JH4287). Full details of the construction of this strain can be found in Table S2.

#### Phage replication assay

Stationary phase cultures of the reporter bacteria were diluted to OD_600_ 0.4 with LB. Aliquots (0.2 mL) were prepared in 1.5 mL tubes and phage stock suspensions were added to a final phage titer of 100-1000 PFU/mL. The infections were carried out at 37°C (30°C for Φ80*p*SU3^+^) with shaking for 2-4 hours and were stopped by the addition of 20 μL of chloroform. After a 10 sec vortex, the lysates were centrifuged (20,000 X *g*, 5 min) and serial diluted. When M9 Glu^+^ was used, *Salmonella* strains were grown to OD_600_ ∼ 0.5 in this medium prior to phage infection.

Phage titer was determined by plaque assay: 10 μL of the dilutions were applied to bacterial lawns of the appropriate reporter strain in technical triplicates. Plaques were enumerated after 16-20 hours of incubation and phage titers (PFU/mL) were calculated for each lysate. To measure the phage input at time 0 (T^0^), the same volume of stock phage suspension was added to 0.2 mL of bacteria-free LB and the titer was determined as described above. The fold-replication for each phage was calculated as the phage titer of the lysate post infection divided by the input phage titer at T^0^. When the phage titer in the lysate was lower than the phage input, the replication was considered to be null (<1-fold). When AHT inducible *tetR-P*_*tetA*_*-bstA* strains were used, AHT (500 ng/mL) or methanol (mock) were added to the diluted bacterial suspension and phages were added after 15 min of incubation at 37°C with aeration.

For replication assays of the coliphages λ, P1 *vir* and Φ80*p*SU3^+^_,_ *E. coli* strains were grown to exponential phase (OD_600_ 0.4) in LB and phages were added as mentioned above. To stimulate infection by these phages, maltose (0.2%), CaCl_2_ (10 mM) and MgSO_4_ (10 mM) were added during the infection and in the lawns of the reporter *E. coli* MG1655. All the phage replication experiments presented were carried out at least twice with biological triplicates.

#### Induction of P22 and BTP1 prophages

D23580 ΔΦ-derived lysogens that carried the different versions of P22 and BTP1 were constructed as detailed in the Table S2. For complementation with the pUC18-derived plasmids (Ap^R^), Ap sensitive lysogens were constructed by the inactivation of the Tn*21*-like element, as described above. The resulting lysogens were grown to stationary phase in LB and the pre-cultures were diluted 100-1000 times in fresh LB and grown to OD_600_ 0.4-0.5, prior addition of Mitomycin C (MitC, 2 μg/mL). The induced cultures were incubated for 3-5 hours at 37°C with aeration and cultures were filter sterilized and serial diluted. The phage titer was measured by plaque assay on the appropriate host strain lawn with technical replicates, as described above. All the prophage induction experiments were carried out at least twice with biological triplicates.

#### Survival assays

For the survival assays, D23580 Δ*tsp-gtrAC* (JH4287), D23580 Δ*tsp-gtrAC bstA*^*STOP*^ (SNW431) or D23580 ΔΦ [P22] (SSO-128) were grown in M9 Glu^+^ to OD_600_ ∼0.5 and two 0.5 mL subcultures were prepared for each culture. The use of D23580 ΔΦ [P22] in these experiments controlled for the effect of lysis from without due to use of high multiplicity of infection (MOI). The strain is a lysogen for WT P22 phage, and therefore is highly resistant to infection by P22-derived phages. P22 Δ*c2* was added at an MOI of 5. The same volume of LB was added to the two remaining subcultures (non-infected controls). Samples were incubated for 15 min at 37°C to allow phage attachment. To stop phage development, the cultures were chilled on ice and bacteria were washed with 0.5 mL of cold PBS. All the samples were serial-diluted in PBS to 10^-6^ and kept on ice. For the measure of survival post-infection, 10 μL of diluted infected or non-infected cultures were applied in technical triplicates on LB agar supplemented with 10 mM EGTA (EGTA was used to minimize secondary infection by free phages). Colony forming Units (CFU) were enumerated and the survival rate, was calculated as the ratio of CFUs in infected cultures divided by the CFUs obtained from non-infected cultures (in %). All the survival experiments were carried out at least twice with biological triplicates.

#### Frequency of lysogeny assays

For the frequency of lysogeny assays, a derivate of phage P22 was used that has the *pid* locus replaced with an *aph* cassette yielding kanamycin resistant lysogens (P22 Δ*pid*::*aph*, SNW490). The *pid* locus has previously been shown to be non-essential in phage P22 and does not establishment of lysogeny (Cenens et al., 2013). D23580 ΔΦ *tetR-P*_*tetA*_*-bstA* (SNW576) cells were grown in 3 mL of LB to OD_600_ ∼0.35. Methanol (mock) or AHT (500 ng/mL, inducer) were added to the cultures and bacteria were incubated to induce BstA for 1 hour at 37°C. 200 μL samples of the bacteria were mixed in triplicate with P22 Δ*pid*::*aph* phage to achieve a MOI of 0.1, and incubated at 37°C for 20 minutes to allow adsorption and ejection of nucleic acids. Cells were pelleted and resuspended in LB media supplemented with 10 mM EGTA to minimize secondary infection by any free phages (along with methanol or AHT) and incubated at 37°C for a further 20 minutes to allow integration and expression of the kanamycin resistance determinant. CFU were enumerated on LB kanamycin. Frequency of lysogeny was determined as the kanamycin resistant CFU/mL divided by the PFU/mL of input phage.

#### Phage DNA detection by Southern Blotting

D23580 ΔΦ *tetR-P*_*tetA*_*-bstA* (SNW576) was grown in 50 mL LB to OD_600_ ∼0.35. The culture was split in two 20 mL sub-cultures and methanol (mock) or AHT (500 ng/mL, inducer) were added to each subculture. Bacteria were incubated to induce BstA for 20 min at 37°C and the phage of interest was added at an MOI of 5. Infections were carried out at 37°C with aeration and total DNA was extracted (Quick-DNA Universal Kit Zymo) from 1.5 mL of culture at 0, 10, 20, 30, 35, 40 and 50 minutes post Infection. Total DNA (100 ng, according to QuBit quantification) was size-separated (2 hours at 100 V in TAE 1X) on a 0.8 % agarose-TAE gel containing Midori Green DNA staining (4 μL for 100 mL gel). One hundred nanograms of non-infected D23580 ΔΦ *tetR-P*_*tetA*_*-bstA* genomic DNA were used as a negative control. DNA was fragmented by exposing the agarose gel to UV light for 5 min on a UV-transilluminator. DNA was denatured by soaking the gel in the Denaturation Solution (0.5 M NaOH, 1.5 M NaCl) for 30 min and then in the Neutralization Solution (1.5 M NaCl, 1 M Tris-HCl, pH 7.6) for 30 min. DNA was transferred on a positively-charged Nylon membrane using the capillary blotting method. Phage DNA was detected with DIG labelled dsDNA probes generated by PCR amplification with MyTaq DNA polymerase (Bioline), buffer, phage DNA and primers (0.4 μM each), in the presence of 0.2 mM dATP, 0.2 mM dCTP, 0.2 mM dGTP, 0.13 mM dTTP and 0.07 mM DIG-11-dUTP. For the 9NA probe a 588 bp PCR fragment was generated with primer pair NW_602 / NW_603 and for the P22/BTP1 probe a 725 bp PCR fragment was generated with primer pair SO-22 / SO-23. The DNA probes were heat-denatured at 95°C for 15 min and the DNA-DNA hybridizations were carried out at 45°C for 16 hours in DIG-Easy Hyb buffer. The washing and immunodetection procedures were carried out, as specified in the DIG Application Manual for Filter Hybridization (Roche) and the chemiluminescence signal was detected using an ImageQuant LAS 4000 imager (GE Healthcare). Prior to DNA transfer onto the membrane, the Midori green-stained DNA was visualized under UV and the resulting image was used as a loading control.

#### Phagemid efficiency of transformation

To avoid a reduction in transformation caused by interspecies DNA modification/restriction interference between *E. coli* and *Salmonella*, all the P22-derived phagemids were first replicated and extracted from *S.* Typhimurium SNW555 before efficiency of transformation assays.

*Salmonella* strains carrying the *tetR-P*_*tetA*_*-bstA* module were grown in 50 mL LBO culture. When OD_600_ ∼0.4 was reached, each culture was split into two 25 mL sub-cultures and methanol (mock) or AHT (inducer) were added to each subculture. Bacteria were incubated for BstA induction during 15 min at 37°C. The cultures were incubated on ice for 5 min and bacteria were washed twice with cold water (25 mL) and were concentrated in 0.1 mL of ice-cold sterile 10% glycerol. The OD_600_ of each electro-competent cell sample was measured by diluting 10 μL of competent cells with 990 μL of 10% glycerol. Cell concentration was adjusted with 10% glycerol for each sample, according to the sample with the lowest OD_600_. The competent cells (20 μL) were mixed with 10 ng (estimated by Qubit) of the P22 phagemids, pP22 (pNAW229) or pP22-*aba* (pNAW230) and the mixture was incubated on ice until electroporation (2.5 KV). Transformation reactions were re-suspended in 1 mL LB or 1 mL LB + AHT (for the *bstA*-induced bacteria) and were incubated for 60 min at 37°C, for recovery. The transformations were diluted (decimal dilution to 10^-5^) in LB or LB+AHT and 100 μL of each dilution (including the non-diluted sample) were spread on LB agar Km or LB agar Km+AHT plates. After incubation at 37°C, the number of Km^R^ transformants was enumerated for each transformation and efficiency of transformation was defined as the number of transformants obtained per ng of phagemid. This experiment was performed with biological triplicates and was repeated twice with LT2 *tetR-P*_*tetA*_*-bstA* (SNW389) and once with D23580 ΔΦ *tetR-P*_*tetA*_*-bstA* (SNW576), giving similar results.

#### Microscopy- general

For all imaging experiments, bacteria were sub-cultured in liquid M9 Glu^+^ media. All images were collected with a wide field Nikon Eclipse Ti-E inverted microscope equipped with an Okolab Cage Incubator warmed to 37°C with Cargille Type 37 immersion oil. A Nikon CFI Plan Apo DM Lambda 100X 1.45 NA Oil objective and a Nikon CFI Plan Apo DM Lambda 20X.75 NA objective were used with Perfect Focus System for maintenance of focus over time. Superfolder GFP, mCherry and SYTOX Orange Nucleic Acid Stain (ThermoFisher) were excited with a Lumencor Spectra X light engine with Chroma FITC (470/24) and mCherry (575/25) filter sets, respectively and collected with a Spectra Sedat Quad filter cube ET435/26M-25 ET515/30M-25 ET595/40M-25 ET705/72M-25 and a Spectra CFP/YFP/mCherry filter cube ET475/20M-25 ET540/21M-25 ET632/60M-25. Images were acquired with an Andor Zyla 4.2 sCMOS controlled with NIS Elements software. For time-lapse experiments, images were collected every 3 minutes (unless specified otherwise) via ND acquisition using an exposure time of 100 ms and 50% or 100% illumination power for fluorescence. Multiple stage positions (fields) were collected using the default engine Ti Z. Fields best representing the overall experimental trend with the least technical artefacts were chosen for publication. Gamma, brightness, and contrast were adjusted (identically for compared image sets) using FIJI (Schindelin et al., 2012). The FIJI plug-ins Stack Contrast (Capek et al., 2006) and StackReg (Thévenaz et al., 1998) were used for brightness matching and registering image stacks.

#### Microscopy- agarose pads

Agarose pads were prepared with 2% agarose and M9 Glu^+^ media, and mounted on MatTek dishes (No. 1.5 coverslip, 50 mm, 30 mm glass diameter, uncoated). Cells (D23580Δ*tsp-gtrAC* (JH4287) or D23580Δ*tsp-gtrAC bstA*^*STOP*^ (SNW431) were grown to log phase (OD_600_ ∼ 0.4) in M9 Glu^+^ at 37°C with shaking (220 RPM), and where required, diluted in fresh M9 Glu^+^ to achieve the desired cell density on the agarose pad. For experiments where all cells were infected (Figure 4A), phage P22 Δ*c2* was added at an MOI of 5. Phage adsorption and initial infection was facilitated by incubation at 37°C with shaking for 10 minutes. Subsequently, infected cells were pelleted at 5000 x g and resuspended in ice-cold PBS to pause phage development. Two microliters of chilled, infected cells were spotted onto opposite sides of an agarose pad (two strains were imaged on the same pad) and inverted onto the MatTek imaging dish. Experimental MOIs were immediately confirmed by CFU and PFU /mL measurement of the cell and phage preparations. Phase-contrast images using the 100X objective were collected every 3 minutes for 3 hours.

Procedures for experiments involving a subset of infected cells (Figure 4C) were identical, except cells infected with P22 Δ*c2 P-mcherry* were washed an additional 4 times in ice-cold PBS to reduce the concentration of un-adsorbed, free phage. In parallel, uninfected cells were washed once in ice-cold PBS. Infected cells were mixed at a ratio of 1:1000 with uninfected cells of the same genotype before being spotted onto the agarose pad. This ratio of uninfected to infected cells was optimized such that in randomly chosen microscopy fields (without prior knowledge of which cells in the field were infected) there was likely to be at least 1 infected cell. Infected cells were retrospectively identified during image analysis by their synchronized lysis within a 10-minute window at the beginning of the microscopy timelapse. For these experiments, phase-contrast and fluorescence images (mCherry) using the 20X objective were collected every 3 minutes for 3 hours.

#### Microscopy- microfluidic infection

The CellASIC ONIX2 system from EMD Millipore with B04A plates was used for microfluidic imaging experiments (Figure S3). Phages used in microfluidic infection experiments shown in Figure S5B (P22 HT or 9NA) were stained with SYTOX Orange Nucleic Acid Stain according to the protocol previously described (Valen et al., 2012). Stained phages washed 4 times in 15 mL M9 Glu^+^ media using Amicon Ultra-15 centrifugal filter units. After staining, the titer and viability of phages were immediately assessed by plaque assay, and once stained, phages were used for no longer than 2 weeks. For use in the microfluidic experiments, SYTOX Orange stained phages were normalized to a titer of approximately 10^10^ PFU/mL. Cells (D23580 *bstA-*_*sf*_*gfp*, SNW403) were grown to early exponential phase (OD_600_ ∼ 0.1) in M9 Glu^+^ at 37°C with shaking (220 RPM) before being loaded into CellASIC B04A plates using the pressure-driven method according to the manufacturer protocol for bacterial cells. The slanted chamber of the plate immobilizes the cells, but allows media to flow continuously. Firstly, cells were equilibrated with constant M9 Glu^+^ media flow for approximately 30 minutes. Secondly, stained phages suspended in M9 Glu^+^ media were flowed over the cells until the majority of cells were infected (typically 10-30 minutes). In the case of P22 HT phage (which exhibits inefficient adsorption to D23580 *bstA-*_*sf*_*gfp* due to the *gtr* locus of prophage BTP), phages were continuously flowed. Finally, M9 Glu^+^ media was flowed over the cells for the duration of the experiment. Microfluidic experiments typically lasted 5 hours, after which time uninfected cells outgrew the chamber. Phase-contrast and fluorescence images were collected every 1.5 minutes for the experiments in Figure S3C.

For the microfluidic imaging experiments shown in Figure S3D, strain SVO251 (S. Typhimurium D23580 ΔΦ *STM1553*::(*P*_*tetA*_*-bstA-*_*sf*_*gfp-frt*) ΔpSLT-BT ΔpBT1 pAW61 (*P*_*BAD*_-*parB-mcherry*) was used. This strain contains the *bstA-*_*sf*_*gfp* fusion construct under the control of the *P*_*tetA*_ promoter. However, this strain lacks the *tetR* gene, and therefore expression of *bstA-*_*sf*_*gfp* is constitutive (not inducible). Additionally, this strain is cured of two natural plasmids that contain native partitioning systems (pSLT-BT and pBT1), and therefore might interfere with the correct function of the ParB-*parS* system used for phage DNA localization. The ParB-mCherry fusion protein is expressed from the pAW61 plasmid (Ap^R^) under the control of the P_BAD_ promoter (induced by L-arabinose). Strain SVO251 was grown in M9 Glu^+^ supplemented with 100 μg/mL ampicillin to maintain the pAW61 plasmid and 0.2% L-arabinose to induce expression of ParB-mCherry. The same supplemented media was used in the microfluidic chamber. Cells were grown to ∼OD_600_ 0.1 before loading into the CellASIC B04A plate as described above. After 15 minutes growth, phage P22 Δ*pid::*(*parS-aph*) [which contains one parS site along with a kanamycin resistance locus, *aph*, in place of the non-essential *pid* locus (Cenens et al., 2013)] diluted to a concentration of 10^8^ PFU/mL (in M9 Glu^+^ amp100 0.2% L-ara) was flowed into the chamber. Phase contrast and red and green fluorescence images were collected every 2 minutes for 4 hours.

#### BstA protein homolog analysis

BstA protein homologs were identified using tblastn (database: non-redundant nucleotide collection) and the HMMER webserver (Potter et al., 2018) (database: Reference Proteomes). The dataset of BstA protein homologs was manually curated to reflect the diversity of taxonomic background harbouring homologs. Evolutionary covariance analysis was done using DeepMetaPSICOV (Buchan and Jones, 2019) at the PSI-PRED server (Kandathil et al., 2019). To analyse the genetic context of BstA homologs, the sequence region 20 kb either side of the homolog (40 kb total) was extracted (BstA 40 kb neighbourhoods). To produce homogenous and comparable annotations, each region was re-annotated using Prokka 1.13 (Seemann, 2014). Additionally, the resulting annotated amino acid sequences were queried against our custom BstA profile-hmm and the Pfam 31.0 database (El-Gebali et al., 2019) with hmmerscan (Eddy, 1998), and the highest scoring significant hit per ORF was considered for the results shown in Figure 2. All the code is available in https://github.com/baymLab/2020_Owen-BstA.

Pairwise identity of homologs in Figure 2B to BstA^BTP1^ was computed using the EMBOSS Needle webserver (Needleman and Wunsch, 1970). BstA homologs were designated “putatively-prophage associated” if annotated genes in the 40 kb neighborhood contained any instance or the word “phage” or “terminase”. For categorization in Figure 2C, homologs were classed as having “high confidence association” if instances of gene annotations including the aforementioned key words occurred both before, and after, the BstA gene within the 40 kb neighborhood (i.e., to account for the possibility that a prophage-independent homolog could co-occur next to a prophage region by chance). Homologs classed as having “low confidence association” had at least one instance of genes whose annotations included “phage” or “terminase” either in the upstream or downstream 20 kb, but not both. Plasmid status was determined from information in the sequence records. The HHpred webserver was used to annotate the putative KilA-N domain (Zimmermann et al., 2018). All homolog neighbourhoods, homolog alignments and sequences is available to download https://github.com/baymLab/2020_Owen-BstA.

### Quantification and statistical analysis

The phage replication, survival rate, efficiencies of transformation and of lysogeny were calculated as mentioned above. The numerical data were plotted and analyzed using GraphPad Prism 8.4.1. Unless stated otherwise in the Figure legends, data are presented as the mean of biological triplicates ± standard deviation. The unpaired t-test was used to compare the groups and statistical significance is indicated on the figures. P values are reported using the following criteria: < 0.0001 = ^∗∗∗∗^, 0.0001 to 0.001 = ^∗∗∗^, 0.001 to 0.01 = ^∗∗^, 0.01 to 0.05 = ^∗^, ≥ 0.05 = ns.

## Data Availability

• All raw data from assays and microscopy reported in this paper will be shared by the lead contact upon request. This paper analyses existing, publicly available data. These accession numbers for the datasets are listed in Table S1. • This paper does not report original code. • Any additional information required to reanalyze the data reported in this work paper is available from the lead contact upon request.

## Supplemental information

Document S1. Figures S1–S6

Table S1. Details of all BstA homologs used in the analysis presented in Figure 2

Table S2. Details of all oligonucleotide sequences, plasmids, bacterial strains, and phages used in this study, related to the STAR Methods

Document S2. Article plus supplemental information
