# Supplementary material for: Prophages encode phage-defense systems with cognate self-immunity
Source: Cell Host Microbe. 2021 Nov 10;29(11):1620–1633.e8. doi: 10.1016/j.chom.2021.09.002 (PMC8585504; doi:10.1016/j.chom.2021.09.002)
Supplement: Document S1. Figures S1–S6 [file mmc1.pdf]

Cell Host & Microbe, Volume *29*

Supplemental information

Prophages encode phage-defense systems with cognate self-immunity

Siân V. Owen, Nicolas Wenner, Charles L. Dulberger, Ella V. Rodwell, Arthur Bowers-Barnard, Natalia Quinones-Olvera, Daniel J. Rigden, Eric J. Rubin, Ethan C. Garner, Michael Baym, and Jay C.D. Hinton

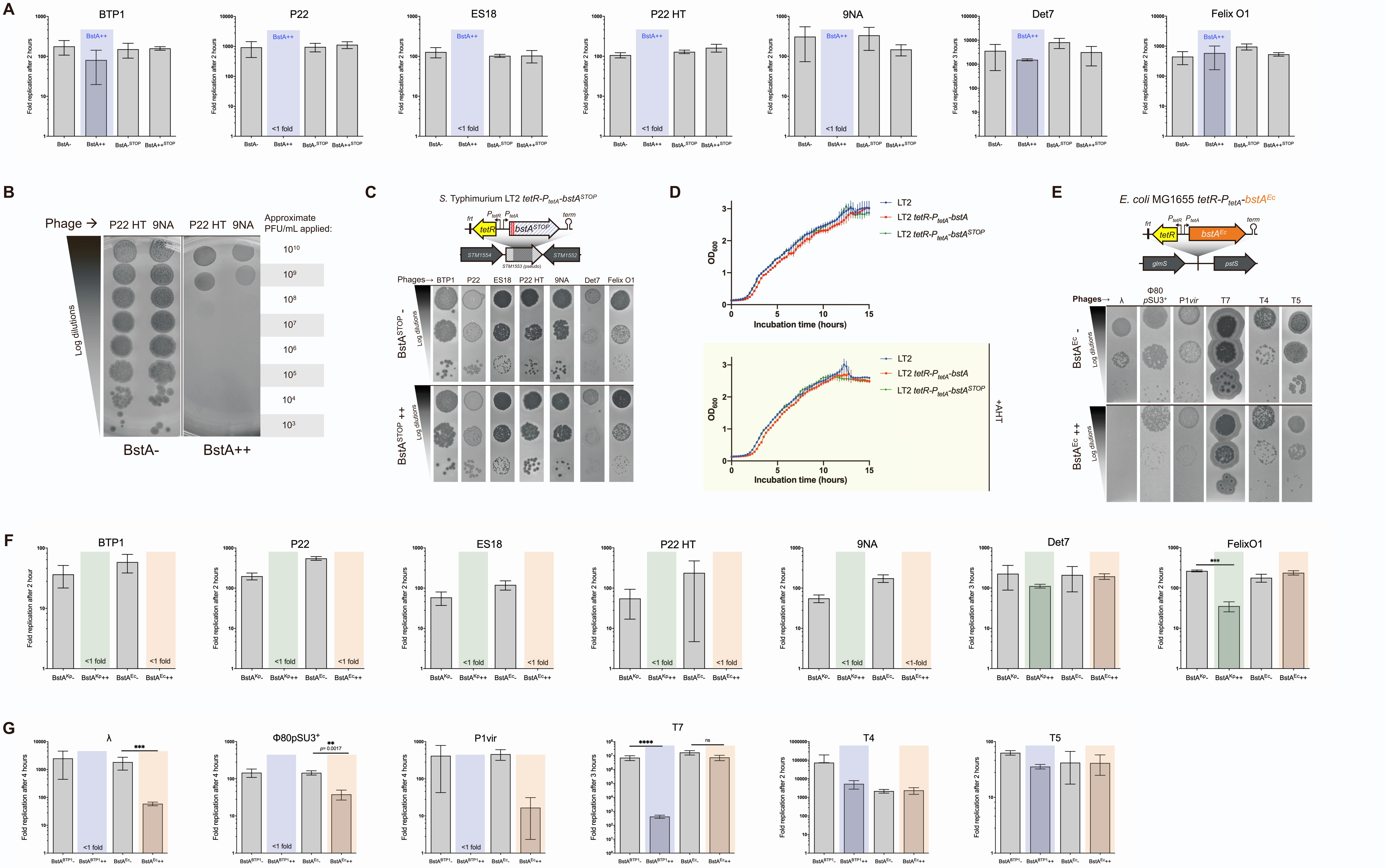

Supplementary Figure 1: The *bstA* locus from prophage BTP1 and homologs from *Klebsiella* and *E. coli* prophages confer phage resistance, related to Figure 1

(A) Replication assays of the indicated phages were carried out with mock-induced (BstA-) or AHT-induced (BstA++) strains LT2 *tetR-P$_{tetA}$-bstA^{BTP1}* (JH4400) or LT2 *tetR-P$_{tetA}$-bstA^{STOP}* (JH4402, carrying two nonsense mutations in *bstA*), as host. Phage replication was measured 2-3 hours post infection and phages were enumerated on lawns of LT2 WT. Phage replication is presented as the mean of biological triplicates ± SD. **(B)** Extended concentration plaque assay of phage P22 HT and 9NA on LT2 *tetR-P$_{tetA}$-bstA^{BTP1}* (JH4400) with mock (BstA-) or AHT induction (BstA++). The approximate PFU/mL **(C)** Nonsense mutations in bstA suppress the BstA-driven anti-phage phenotype. Plaque assays were carried out with the indicated phages on mock- or AHT-induced lawns of LT2 *tetR-P$_{tetA}$-bstA^{STOP}* (JH4402). (**D**) Optical density growth curves (OD600) of LT2 WT, LT2 *tetR-P$_{tetA}$-bstA^{BTP1}* (JH4400) or LT2 *tetR-P$_{tetA}$-bstA^{STOP}* (JH4402, carrying two nonsense mutations in *bstA*)) with and without AHT induction. No cellular toxicity (as measured by reduced culture growth) was associated with *bstA^{BTP1}* or *bstA^{STOP}* expression. (**E**) BstA^{Ec} confers phage resistance to *E. coli*. Plaque assay were carried out with the indicated phages applied on a lawn of mock- or AHT-induced MG1655 *tetR-P$_{tetA}$-bstA^{Ec}* (JH4414). (**F**) BstA^{Kp} and BstA^{Ec} confer phage resistance in *S.* Typhimurium. (**G**) BstA^{BTP1} and BstA^{Ec} confer phage resistance in $^{E. coli}$. Phage replication assays were carried out with mock-induced (BstA-) or AHT-induced (BstA++) cultures of LT2 *tetR-P$_{tetA}$-bstA^{Ec}* (JH4408), LT2 *tetR-P$_{tetA}$-bstA^{Kp}* (JH4404), MG1655 *tetR-P$_{tetA}$-bstA^{BTP1}* (JH4410) or MG1655 *tetR-P$_{tetA}$-bstA^{Ec}* (JH4414), infected with the indicated phage. LT2 or MG1655 WT lawns were used for phage enumerate 2-3 hours post infection. Phage replication is presented as the mean of biological triplicates ± SD. When replication difference between induced and non-induced cultures was lower than one order of magnitude, groups were compared using unpaired two-tailed Student t-test and P values and significance are indicated by *,**,*** or ns (not significant).

A

B

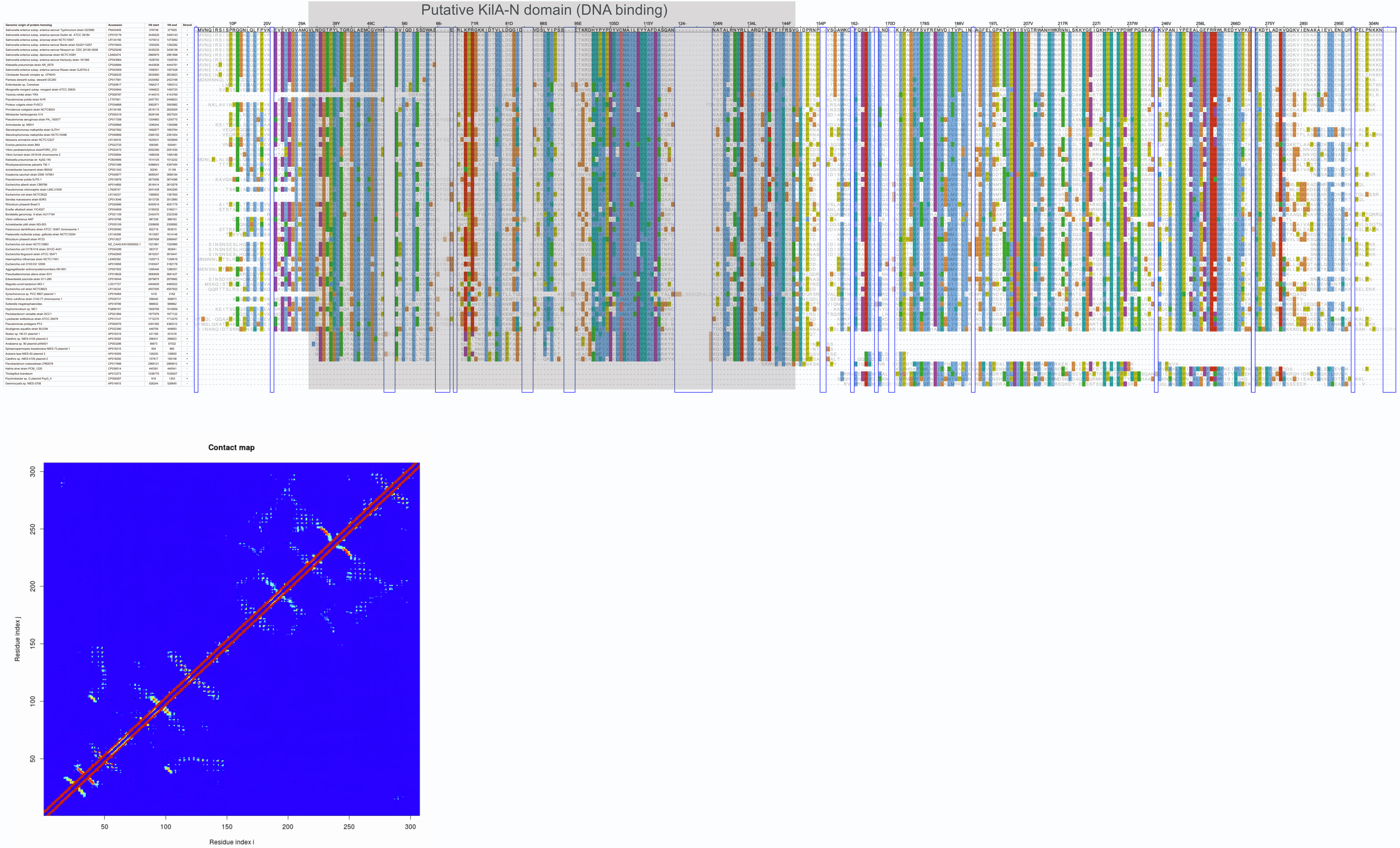

Supplementary Figure 2: Extended alignment of BstA protein homologs and predicted BstA contact map, related to Figure 2

(A) Blue boxes indicate columns which are gaps relative to the reference sequence (top row, BstA from S. Typhimurium D23580), and are collapsed in the alignment shown in Figure 2. Grey box indicates the position of the putative KilA-N domain. **(B)** Predicted contact map derived by evolutionary covariance analysis of BstA by DeepMetaPSICOV. The confidence of a pair of residues x and y interacting is indicated by the colour of the cell at position x, y (and by mirroring at position y,x) with red indicating high probability of interaction and dark blue indicating low probability. The map suggests that residues 1-~155 and ~156-end form folded domains that interact very little.

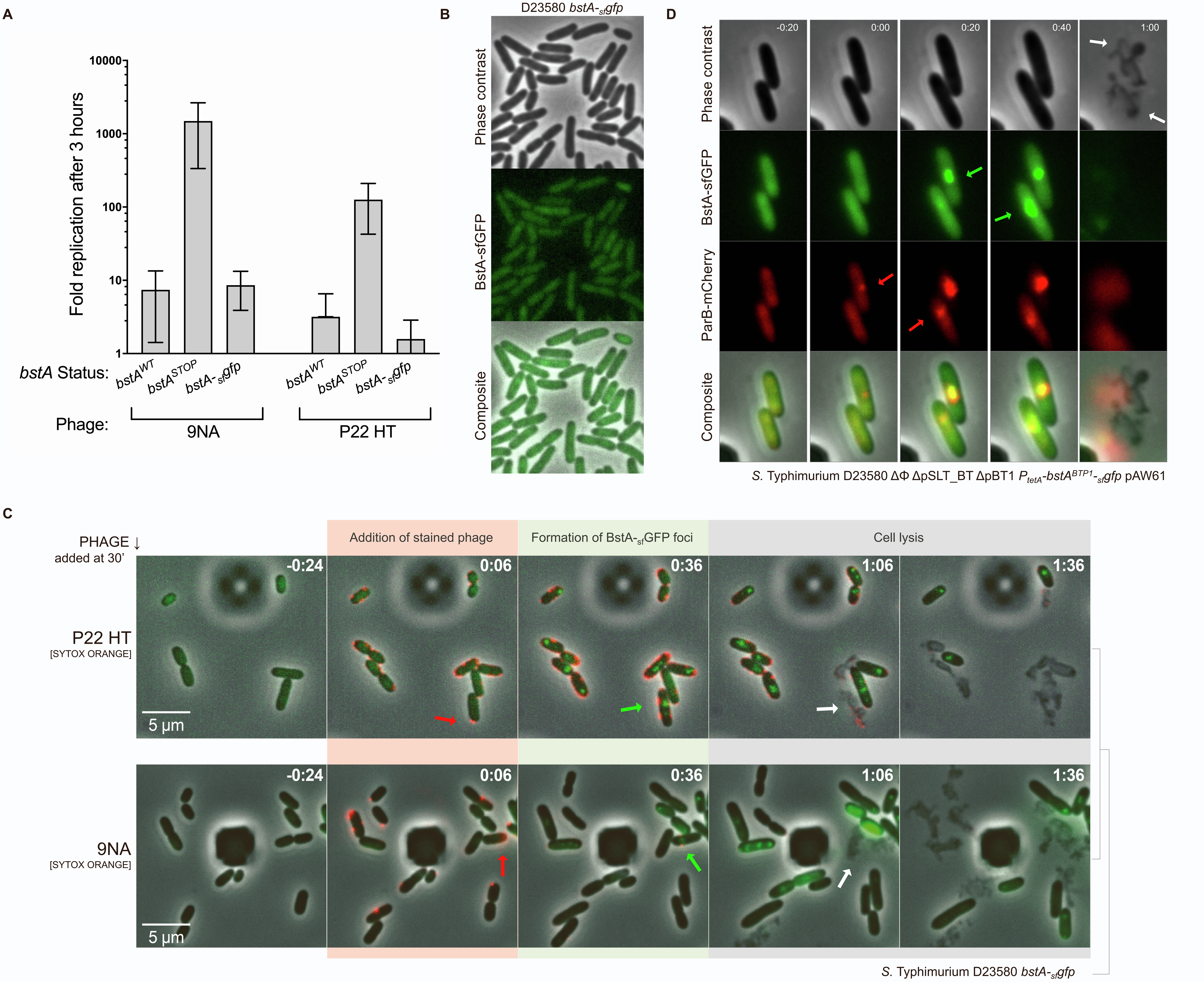

Supplementary Figure 3: BstA protein responds dynamically to phage infection and colocalises with phage DNA, related to Figure 4 and Figure 6

A translational fusion of BstA to $_{sf}$GFP protein (D23580 *bstA-$_{sf}$gfp*, SNW403) was constructed to track the location of BstA protein inside cells. **(A)** C-terminal Superfolder GFP (sfGFP) fusion to BstA does not impair the BstA anti-phage activity. Replication assays were carried with the indicated phages on strains carrying the WT *bstA* (*bstA^{WT}*, the defective *bstA^{STOP}* version (strain SSO-78) or the BstA-$_{sf}$GFP fusion strain (*bstA-$_{sf}$gfp*, strain SNW403)). Phage replication was measured 3 hours post-infection and plaques were enumerated on lawns of D23580 Δ*tsp-gtrAC bstA^{STOP}* (SNW431). Phage replication is presented as the mean of biological triplicates ± SD. **(B)** I n the absence of phage infection, GFP signal is diffuse within the cell cytoplasm, suggesting no specific sub-cellular localisation**.** **(C)** A microfluidic growth chamber was used to observe the behaviour of BstA protein during phage infection, capturing images every 1.5 minutes. A time series of representative fields are presented as composite images (phase contrast, green and red fluorescence are overlaid). Cells were first grown for a period in the chamber (immobilised by the angle of the chamber ceiling) with constant flow of M9 Glu^{+} media (Methods). Fluorescently-labelled phages P22 HT or 9NA (stained with SYTOX Orange resuspended in M9 Glu^{+} media , Methods) were then added to the cells, and can be seen adsorbing to cells as red fluorescent puncta (red arrows). For comparative purposes, timestamps are synchronised to the point at which phage were first observed adsorbing to cells. Typically around 20 minutes after initial observation of phage infection, BstA proteins formed discrete and dynamic foci within the cells (green arrows). Cells then proceeded to lyse (white arrows), consistent with previous microscopy data in Figure 4. Videos of the time series are presented in Supplementary Video 3. **(D)** A microfluidic growth chamber was used to co-localise BstA protein and the DNA of the infecting phage. Prophage-free and plasmid-free SVO251 cells (expressing the BstA-$_{sf}$GFP fusion and a ParB-mCherry fusion protein) were grown for a period of 15 minutes before P22 Δ*pid*: :(*parS-aph*) phages were flowed across the cells. The ParB-mCherry fusion protein oligomerises at the *parS* site on the infecting phage chromosome, so that mCherry foci indicate the subcellular location of phage DNA (red arrows). To facilitate comparison, the time stamp was set to zero at the first observation of mCherry foci (i.e. the earliest detectable event of phage infection). BstA foci formed directly after appearance of mCherry foci (green arrows), and the merging of the image (composite) confirmed an overlap of the foci, consistent with the co-localisation of BstA foci with infecting phage DNA. Cells proceeded to lyse (white arrows). Time is indicated as h:m, and videos of the time series are presented in Supplementary Video

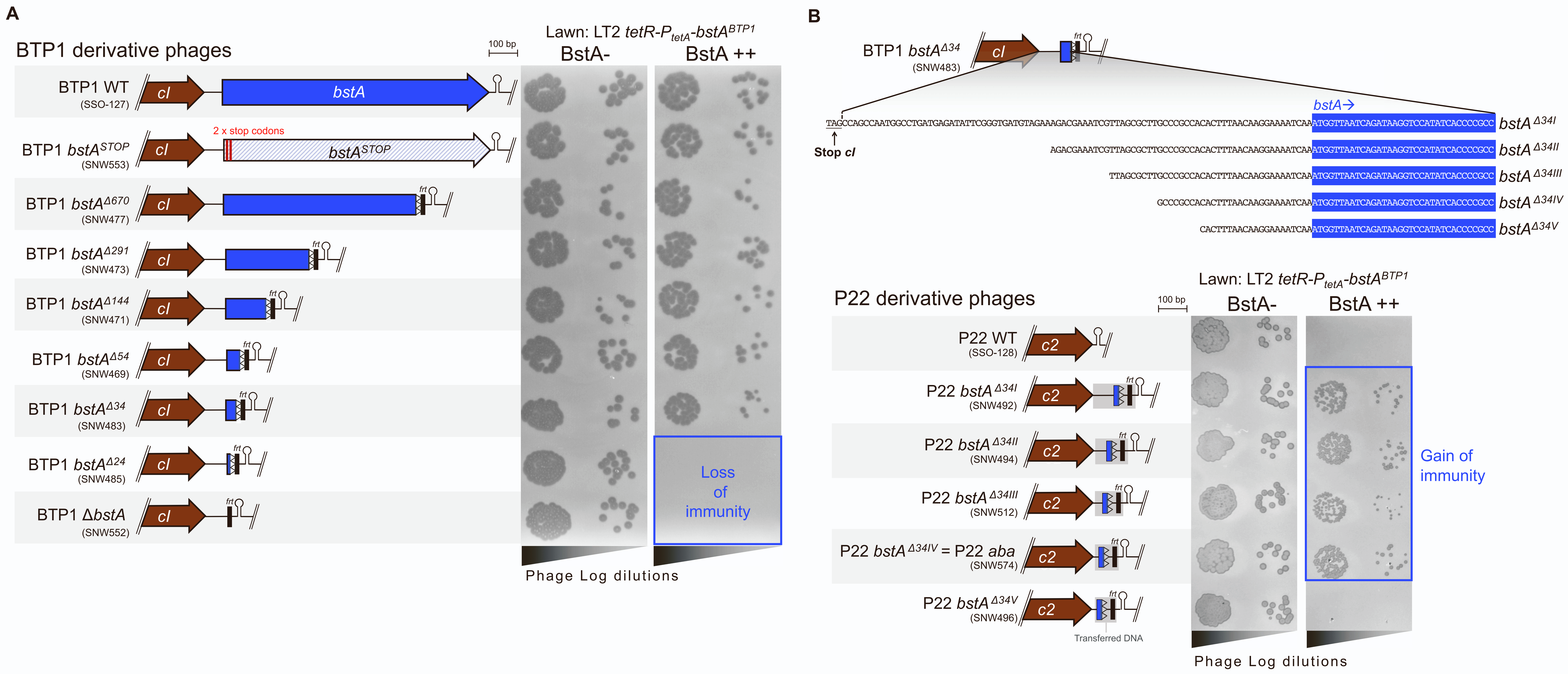

Supplementary Figure 4: Identification of the anti-BstA (*aba*) factor, related to Figure 5

(A) BTP1 *bstA* locus truncations revealed the location of the anti-*bstA* fragment *aba*. The *bstA* truncations are indicated for each BTP1 variant. The *bstA*$^{\Delta}$ allelic numbering corresponds to the number of base pairs remaining from the *bstA* ATG start. **(B)** Transfer of the *bstA*$^{\Delta 34}$ fragment in P22 confers BstA-immunity. Fragments *bstA*$^{\Delta 34I}$- *bstA*$^{\Delta 34V}$ are indicated. The donor lysogen strain for each BTP1 and P22 variant is indicated in brackets. The residual scar sequence of pKD4 is indicated by *frt* and hairpins represent Rho-independent terminators. Plaque assays were performed with the indicated BTP1/P22-derived phages on lawns of mock-induced (BstA-) or AHT-induced (BstA++) LT2 *tetR*-P_{tetA}-*bstA*^{BTP1} (JH4400).

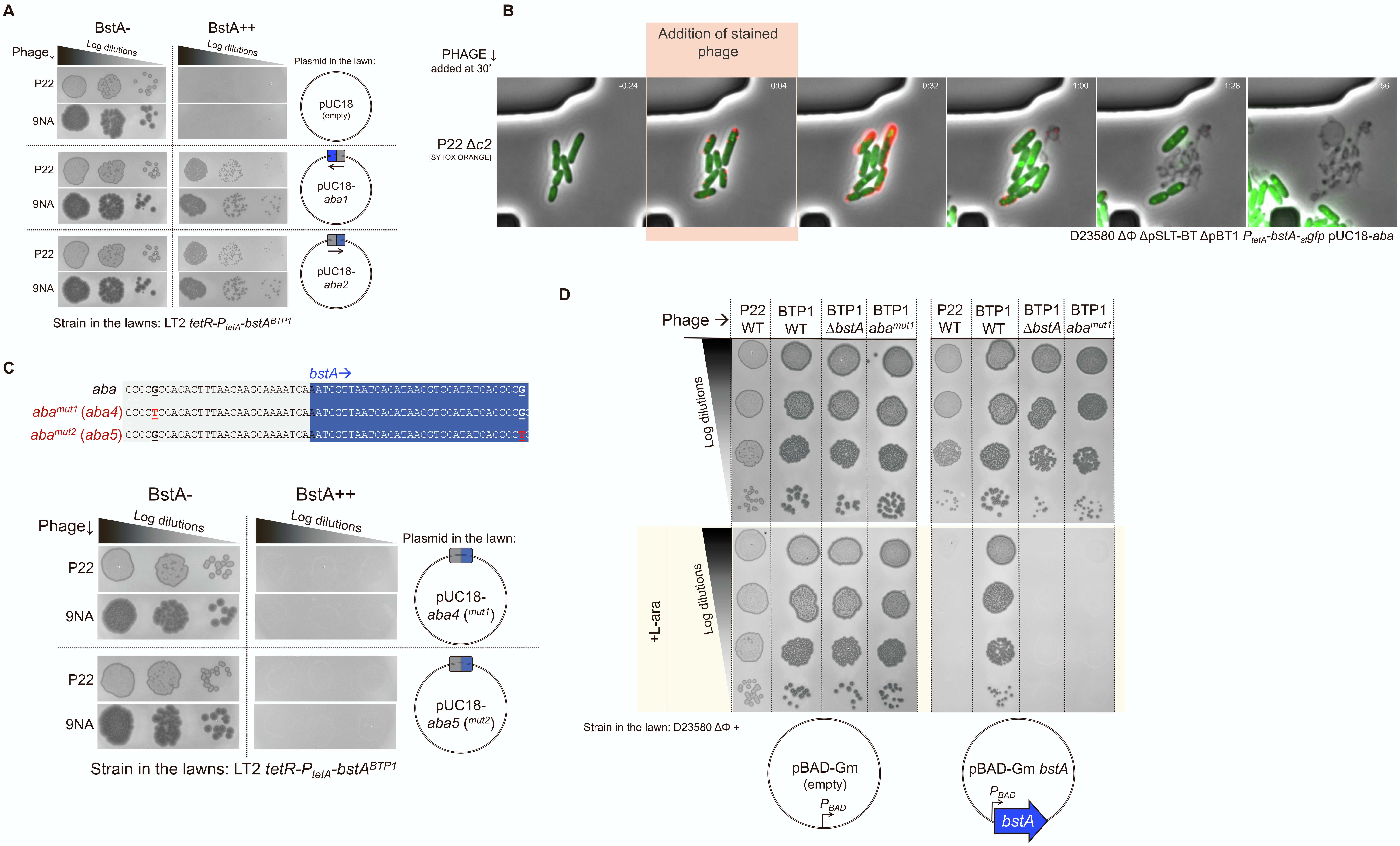

Supplementary Figure 5: Multiple copies of *aba* DNA suppresses BstA activity in trans but do not affect protein localization dynamics, and mutations in the terminal direct repeat motifs of *aba* ablate its function in trans and during phage infection (related toe Figure 5 and Figure 6)

(A) The *aba* sequence was cloned into the pUC18 plasmid in either orientation, and the plasmids were transformed into LT2 *tetR-P_{tetA}-bstA^{BTP1}* (JH4400). Plaques assays were performed with phage P22 and 9NA applied on lawns of mock-induced (BstA-) or AHT-induced (BstA++) LT2 *tetR-P_{tetA}-bstA^{BTP1}* (JH4400), transformed with the indicated plasmid. Both plasmids (pUC18-*aba1* and pUC18-*aba2*) supressed BstA activity and permitted plaque formation by P22 and 9NA in the presence of BstA. **(B)** A microfluidic growth chamber was used to observe the behaviour of BstA protein during phage infection in the presence of the pUC18-*aba* plasmid, capturing images every 4 minutes. A time series of representative fields are presented as composite images (phase contrast, green and red fluorescence are overlaid). Cells (D23580 ΔΦ ΔpSLT-BT ΔpBT1 *P_{tetA}-bstA-$_{sf}$gfp* pUC18-*aba*, SVO254) were first grown for a period in the chamber (immobilised by the angle of the chamber ceiling) with constant flow of M9 Glu^{+} amp100 media (Methods). Fluorescently labelled phage P22 Δ*c2* (stained with SYTOX Orange resuspended in M9 Glu^{+} media, Methods) were then added to the cells. For purposes of comparison, timestamps are synchronised to the point at which phage are first observed adsorbing to cells. Localisation of BstA proteins into foci proceeding cell lysis was conserved in the presence of the pUC18-*aba* plasmid. Mutations in the terminal direct repeat motifs of abaBTP1 ablate its function in trans and during phage infection
(C) The assay shown in Supplementary Figure 5A was repeated but with mutated versions of the *aba* sequence. When a single nucleotide mutation was made in either of the terminal CCCGCC motifs, the pUC18 *aba* plasmids could not rescue the replication of P22 or 9NA in the presence of BstA. **(D)** BTP1 phage carrying the *aba^{mut1}* mutation (which does not alter the coding sequence of the *bstA* gene), were challenged against cells expressing BstA from an arabinose-inducible promoter (P_{BAD}) on a plasmid (plasmid pNAW254). Phages BTP1 WT, P22 WT and BTP1 Δ*bstA* (in which *aba*-mediated BstA immunity is present, absent, and synthetically absent, respectively) were included as comparators. BTP1 *aba^{mut1}* plaques normally in the absence of BstA (in cells carrying the empty plasmid, or cells harbouring the *bstA* plasmid without +L-ara inducer). BTP1 *aba^{mut1}* is unable to form plaques on cells expressing BstA, and is inhibited to the same degree as phage P22 (entirely lacking *aba*) , and BTP1 Δ*bstA* (in which half of the *aba* sequence is deleted).

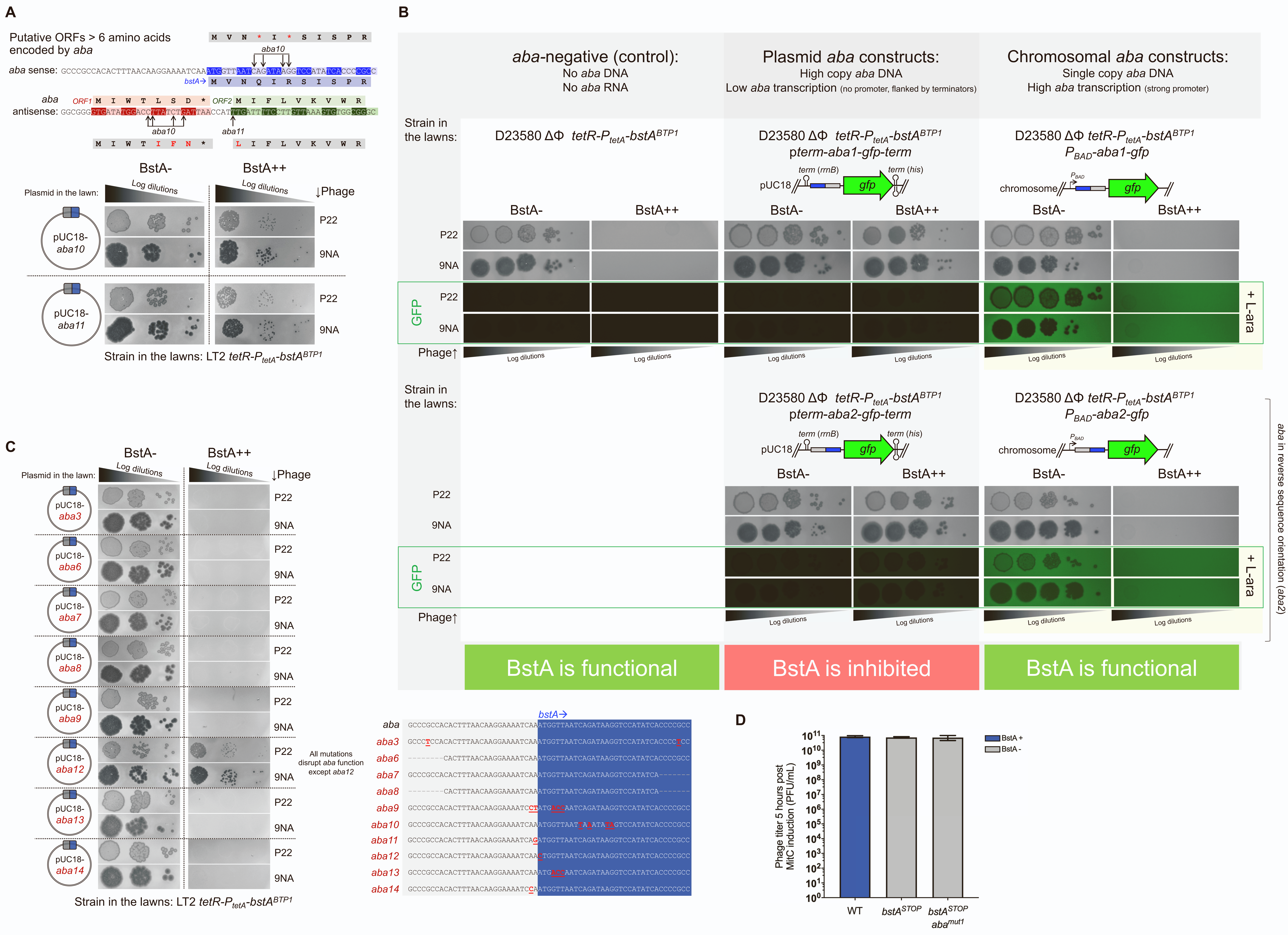

Supplementary Figure 6: *aba* functions as a DNA element, and its function is highly sensitive to sequence mutations (related to Figure 5 and Figure 6)

(A) Analysis of the *aba* sequence: putative open reading frames (ORFs) encoding for peptides longer that 6 amino acids are shown. All the ATG, TTG or GTG triplets were considered as putative start codons. The mutations of the functional *aba10* and *aba11* and the resulting mutated residues (in red) in the putative ORFs are indicated. Stop codons are indicated by *. Non-synonymous mutation in the putative ORFs does not disrupt *aba* suppression of BstA, therefore *aba* is unlikely to function via a peptide. **(B)** Transcription of *aba* does not counteract the BstA anti-phage activity. The *aba* sequence followed by a *gfp* reporter gene were inserted into pUC18 in both directions without promoter and flanked by terminators (*rrnB* terminator upstream and the bidirectional histidine operon terminator downstream). Secondly the *aba* sequence was integrated in both directions in the chromosome of strain D23580 ΔΦ *tetR-P_{tetA}-bstA^{BTP1}* (strains SNW645 and SNW646), downstream of the native arabinose-inducible promoter P_{BAD} (bent arrow) controlled by the AraC regulatory protein. Plaque assays were performed with the indicated phages and strains in the presence or absence of L-arabinose (0.2%). There was very little transcription of the *aba* plasmid constructs as indicated by the lack of *gfp* signal, but the constructs suppressed the activity of BstA. Induction of the transcription of chromosomal *aba* by L-arabinose did not suppress the activity of BstA (P22 and 9NA phage plaquing is inhibited). **(C)** Further mutational disruption was made to the *aba* sequence cloned into pUC18 plasmids, to identify regions of the sequence that are essential for *aba* function. All mutations to pUC18 *aba* were non-functional (i.e. not capable of BstA suppression) except for the *aba12* derivative. Plaques assays were performed with phage P22 and 9NA applied on lawns of mock-induced (BstA-) or AHT-induced (BstA++) LT2 *tetR-P_{tetA}-bstA^{BTP1}* (JH4400), transformed with the indicated plasmid. **(D)** The *aba^{mut1}* mutation has no effect on prophage induction in the absence of BstA protein expression. Prophage induction was measured in strain D23580 ΔΦ lysogenized with BTP1 WT (SSO-127), BTP1 *bstA^{STOP}* (SNW553) and BTP1 *bstA^{STOP} aba^{mut1}* (SNW660) after 5 hours post induction with Mitomycin C (MitC). Data are presented as the mean of biological triplicates ± SD. Prophage BTP1 WT has a functional BstA protein.
